# Double-headed nucleosides: Synthesis and applications

**DOI:** 10.3762/bjoc.17.98

**Published:** 2021-06-08

**Authors:** Vineet Verma, Jyotirmoy Maity, Vipin K Maikhuri, Ritika Sharma, Himal K Ganguly, Ashok K Prasad

**Affiliations:** 1 Bioorganic Laboratory, Department of Chemistry, University of Delhi, Delhi-110 007, India; 2 Department of Chemistry, St. Stephen’s College, University of Delhi, Delhi-110 007, India; 3 Department of Biophysics, Bose Institute, P1/12 CIT Scheme VIIM, Kolkata-700 054, India

**Keywords:** acyclic double-headed nucleosides, bicyclic double-headed nucleosides, furanosyl double-headed nucleosides, modified nucleosides, pyranosyl double-headed nucleosides

## Abstract

Double-headed nucleoside monomers have immense applications for studying secondary nucleic acid structures. They are also well-known as antimicrobial agents. This review article accounts for the synthetic methodologies and the biological applications of double-headed nucleosides.

## Introduction

Nucleosides are the constructional subunits of deoxyribonucleic acids (DNA) or ribonucleic acids (RNA), which contain either a purine or pyrimidine nucleobase and a furanosyl moiety of pentose sugars, 2′-deoxyribose or ribose [1–2]. Nucleotides are constituted by addition of a phosphate group at the 5′-position of the nucleosides and these monomeric units polymerize to construct nucleic acids (DNA or RNA). These macromolecules preserve and express genetic information in all living cells and viruses. Modified nucleosides are a class of organic compounds which are unnatural and have an altered/substituted nucleobase and/or a modified pentose sugar [3–4]. The synthetic accessibility of these organic molecules encouraged researchers to prepare sugar-modified nucleosides [5–6] and nucleobase-modified nucleosides [7–8]. Modified nucleoside monomers comprising more than one nucleobase are called double-headed nucleosides [9–10]. A thorough literature search regarding double-headed nucleosides disclosed that these modified nucleosides were constituted with any two naturally occurring nucleobases, i.e., adenine, guanine, thymine, uracil, and cytosine [9–10] or one naturally occurring nucleobase and one heterocyclic/carbocyclic moiety either attached directly to the sugar or via a linker. Further modifications were introduced by the substitution of some of the naturally occurring nucleobases by halogens or alkyl groups. On the other hand, a variety of heterocyclic/carbocyclic moieties were considered as the head of these modified nucleosides. The heterocyclic structures which were found to be attached to these double-headed nucleosides include triazolophthalazine [11], 4,6-di-*tert*-butylbenzoxazole [12], mesitylisoxazole [13], 5-trimethylsilyl-1,2,3-triazole [14], 1-pivaloyloxymethyl-1*H*-1,2,3-triazole [15], 1,3,4-oxadiazino[6,5-*b*]indole [16], 6,7-dihydro-6-oxo-5*H*-1,2,4-triazolo[3,4-*b*][1,3,4]thiadiazine [17], 1,2,4-triazino[5,6-*b*]indole [18], 1,3,4-thiadiazoline [19], 1,3,4-oxadiazoline [19], 1,2,4-triazoline [19], 3-mercapto-1*H*-1,2,4-triazole [20], 1,3,4-oxadiazole-2(3*H*)-thione [20], 4-amino-5-mercapto-4*H*-1,2,4-triazole [20], and 1,2,4-triazolo[3,4-*b*](1,3,4)-thiadiazole moieties [21]. Additionally, selected examples of double-headed nucleosides comprising aromatic/polyaromatic/carbocyclic moieties such as phenyl [13–15,22], pyrene [23–25], adamant-1-yl [24], cholesteryl [24], perylen-3-yl [24], 4-(*tert*-butyldimethylsilyloxy/hydroxy)phenyl [26], 3/4-(*N*-((dimethylamino)methylidene)aminosulfonyl)phenyl [26–27], and sulfonamido-substituted benzothiazole [28] attached as an additional head are also reported in this review article. Literature data revealed that most of the double-headed nucleosides have the first nucleobase attached to the anomeric carbon of the pentofuranosyl/hexopyranosyl sugar moiety and an additional nucleobase/heterocyclic/carbocyclic moiety attached either directly or through a linker to any carbon of the sugar moiety either by C–N or C–C bonds. However, in case of base to base double-headed nucleosides, the additional nucleobase/substituent or unsubstituted phenyl moiety/polyaromatic moiety/carbocyclic moiety/heterocyclic moiety is attached to the first nucleobase with/without a linker. Whereas, all the acyclic double-headed nucleosides had natural nucleobases or heterocyclic moieties attached at the terminal carbons only.

Double-headed nucleosides are synthetically derived nucleoside scaffolds that are known to impact significantly secondary structures in nucleic acids [29]. Some oligonucleotides containing a particular double-headed nucleotide monomer have been found to form a three-way junction structure with a hairpin loop and two flanking sequences [30–31]. Moreover, these nucleotides have been found to orient the additional nucleobase towards the core of the duplex to participate in Watson–Crick base pairing [32–34]. The incorporation of the double-headed nucleosides into oligonucleotides followed by their duplex formation studies against complimentary oligonucleotide strands had described a very selective zipper-interaction [35], whereas a relative stabilization was observed due to stacking of these additional nucleobases across the minor groove [31]. The biological activity of the acyclic double-headed nucleosides was assessed through in vitro studies on Gram-positive bacteria *Staphylococcus aureus*, *Listeria inovanii* and Gram-negative bacteria *Klebsiella pneumoniae*, *Salmonella* sp., and *Escherichia coli* [20]. Triazolyl double-headed nucleosides showed efficacy against eosinophil-derived neurotoxin, which is an eosinophil secretion protein and a member of the Ribonuclease A (RNase A) superfamily [36]. Double-headed nucleosides were also found to be active against orthopox viruses, vaccinia virus, and cowpox virus under in vitro conditions [11], whereas few double-headed nucleoside analogues showed a moderate cytostatic activity against human cervix carcinoma HeLa cells [37].

It is pertinent to mention that Sharma et al. [29] have reviewed the double-headed nucleotides in the recent past with a focus on their effects in nucleic acid duplexes and other secondary structures. Herein, we focused on the synthetic protocols used for accessing a variety of double-headed nucleoside monomers. Thus, this review is the comprehensive compilation of the synthetic protocols available for the production of double-headed nucleoside monomers and their applications. For better overview the review has been structured based on the types of the sugar moiety of the nucleoside and the position of the attachment of the additional base, either directly or through a linker on the sugar.

## Review

### Furanosyl double-headed nucleosides

**Figure 1:**
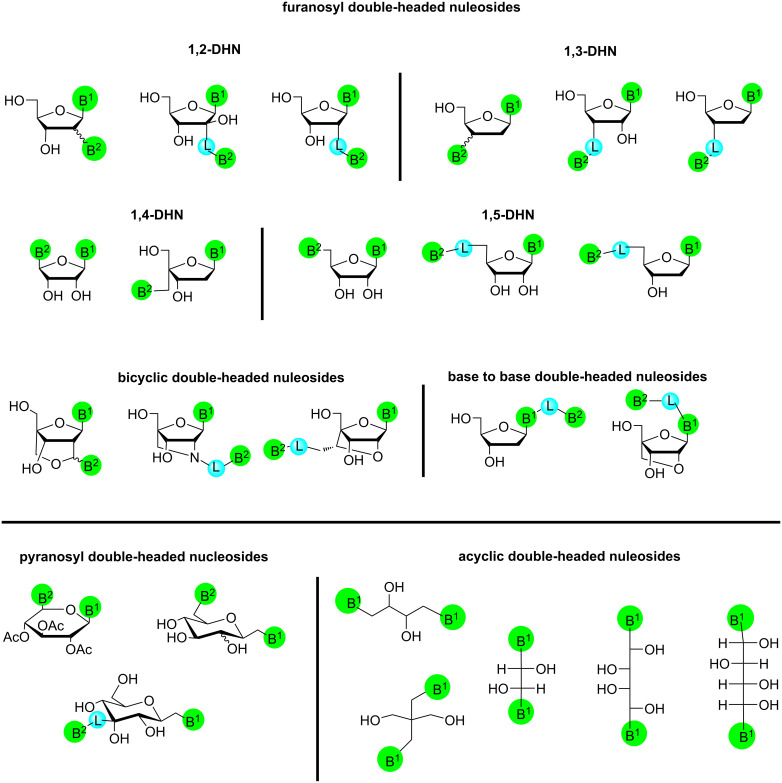
Double-headed nucleosides. B^1^ and B^2^ = nucleobases or heterocyclic/carbocyclic moieties; L = linker.

Based on literature reports most of the double-headed nucleosides comprised a pentofuranosyl sugar moiety. Various synthetic methodologies have been developed for the introduction of the additional nucleobase/heterocyclic system directly or via a linker at the C-2′/C-3′/C-4′/C-5′ position of the pentofuranosyl moieties. We have categorized the double-headed furanosyl-nucleosides depending on the position of the attachment of the additional nucleobase/heterocyclic system at the particular carbon of the pentofuranosyl moiety of the nucleoside (Figure 1).

#### 1,2-Furanosyl double-headed nucleosides

Herein, all nucleosides comprising furanosyl ring structures are included, with the first nucleobase attached to the C-1′ position and the second nucleobase introduced at the C-2′ position either with or without a linker (Figure 1).

**Scheme 1:**
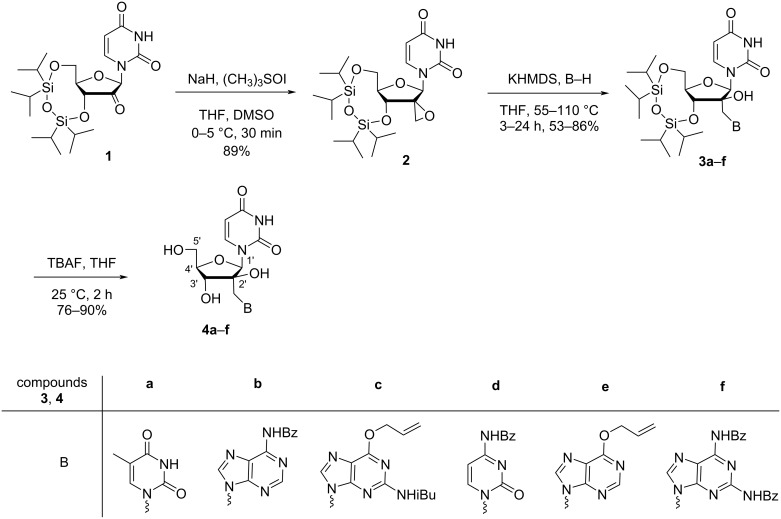
Synthesis of 2′-(pyrimidin-1-yl)methyl- or 2′-(purin-9-yl)methyl-substituted double-headed nucleosides **4a–f** of arabinofuranosyluracil.

Nielsen and co-workers [38–39] synthesized 2′-(pyrimidin-1-yl)methyl- or 2′-(purin-9-yl)methyl-substituted double-headed nucleosides **4a**–**f** of arabinofuranosyluracil. The convergent synthesis of the double-headed nucleosides was achieved from uridine, which was first converted to the 3′,5′-(1,1,3,3-tetraisopropyldisiloxan-1,3-diyl)-protected (TIPDS) ketonucleoside **1** following a standard procedure [40]. The subsequent Corey–Chaykovsky epoxidation [41] of 2′-ketonucloside **1** with trimethylsulfoxonium iodide in DMSO afforded the spironucleoside **2**, which in turn was converted to the TIPDS-protected 2′-(pyrimidin-1-yl)methyl-/2′-(purin-9-yl)methylarabinofuranosyluracil derivatives **3a–f** by nucleophilic epoxide ring opening with thymine, *N*-benzoyladenine, 6-*O*-allyl-*N*-isobutyrylguanine, *N*-benzoylcytosine, 6-*O*-allylhypoxanthine or *N*,*N*-dibenzoyldiaminopurine in 53 to 83% yield. The desilylation of the nucleosides **3a**–**f** with tetrabutylammonium fluoride in tetrahydrofuran (THF) led to the formation of six different double-headed nucleosides **4a**–**f** (Scheme 1) [38–39].

The synthesized double-headed nucleosides **4a**,**b** were dimethoxytritylated (DMTr), phosphitylated, and incorporated into DNA oligonucleotides using the standard automated phosphoramidite method. The UV-based melting temperature (*T*_m_) of hybrids of the modified oligonucleotides with complementary DNA strands were studied. The analysis of the melting temperature of the duplex and extensive molecular dynamics studies revealed that the synthesized double-headed nucleotides behave as functional dinucleotide mimics and hybridize with complementary targets neatly with their Watson–Crick faces compatible with natural DNA [39].

**Scheme 2:**
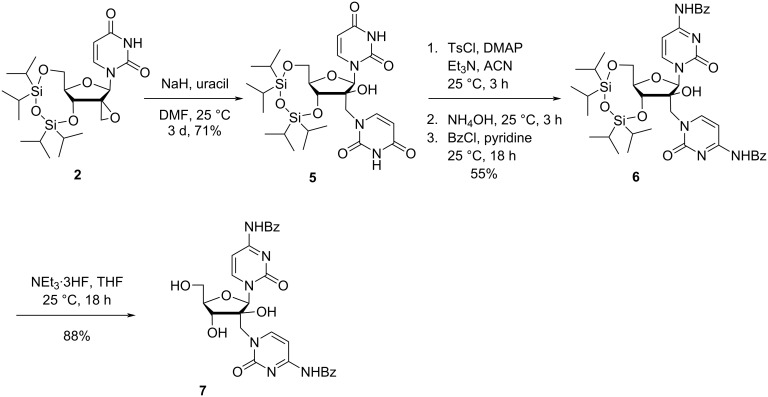
Synthesis of double-headed nucleoside **7** having two cytosine moieties.

Nielsen and co-workers [42] additionally synthesized 2′-(*N*-benzoylcytosin-1-yl)methylarabinofuranosyl-*N*-benzoylcytosine (**7**) from uridine using a similar methodology. Thus, the nucleophilic epoxide ring opening in spironucleoside **2** with uracil in DMF in a *N*^1^-regioselective manner afforded the TIPDS-protected double-headed nucleoside **5** having two uracil bases (the additional uracil being attached through a methylene linker to the 2′-position of arabinouracil). Subsequently, the two uracil bases of the TIPDS-protected double-headed nucleoside **5** were converted to *N*-benzoylated cytosines in a three-step one-pot procedure in 55% yield. For this conversion, the carbonyl group at the 4-position of uracil was first activated by tosylation, which was followed by conversion to the amine upon reaction with ammonia and protection of the newly introduced amino group with benzoyl chloride to afford the double-headed nucleoside **6**. The removal of the silyl protecting group with NEt_3_·3HF in THF yielded 2′-(*N*-benzoylcytosin-1-yl)methyl-arabinofuranosyl-*N*-benzoylcytosine (**7**, Scheme 2) [42].

The double-headed nucleoside **7** was dimethoxytritylated and phosphitylated following the standard procedure and incorporated into oligonucleotides to study its effects on duplex stability. The single incorporation in oligonucleotides and study of the melting temperature (*T*_m_) of its duplex hybridized with a complementary DNA strand revealed an increase in *T*_m_ by 4 °C with respect to the normal duplex. This indicated the participation of both nucleobases of the double-headed nucleotides in Watson–Crick base pairing. The same group also showed that a multiple incorporation of the double-headed nucleotide is also tolerated, but the double-headed nucleotides with the present design were not suitable as triplex-forming oligonucleotides [42].

**Scheme 3:**
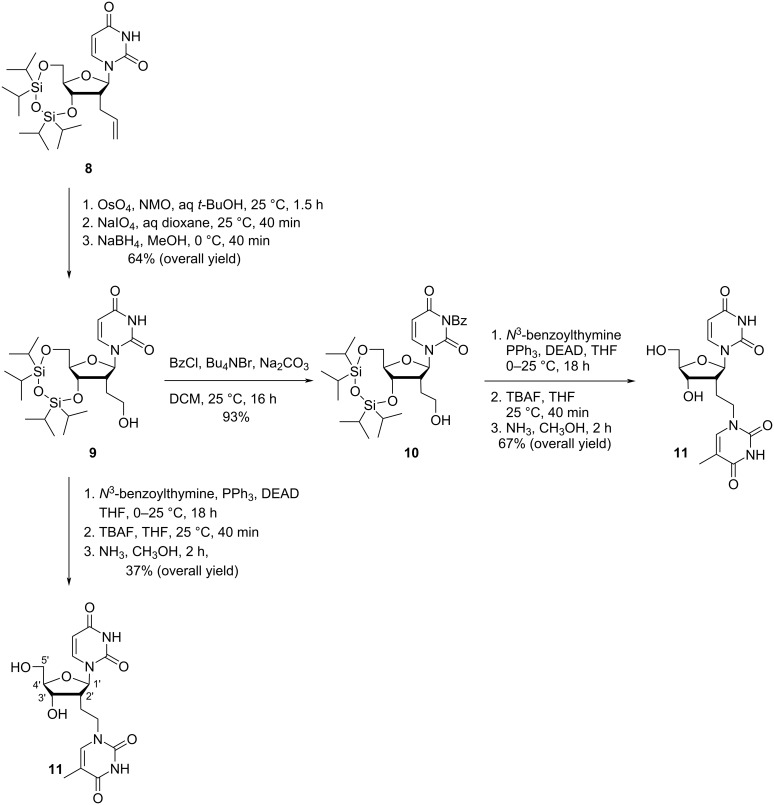
Synthesis of double-headed nucleoside 2′-deoxy-2′-*C*-(2-(thymine-1-yl)ethyl)-uridine (**11**).

Pedersen and Nielsen [35] synthesized a double-headed nucleoside with two different nucleobases, i.e., 2′-deoxy-2′-(thymine-1-yl)ethyluridine (**11**) (Scheme 3). The oxidative cleavage of the allyl group in TIPDS-protected 2-allyl-2-deoxyuridine **8** gave the TIPDS-protected hydroxynucleoside **9** as key intermediate. The treatment of **9** with benzoyl chloride under suitable conditions to selectively protect the 3-NH group of the uracil moiety afforded *N*^3^-benzoyluridine (**10**). The reaction of TIPDS-protected hydroxynucleoside **9** with *N*^3^-benzoylthymine under Mitsunobu reaction conditions, followed by deprotection with TBAF and aqueous methanolic ammonia resulted in the formation of 2′-deoxy-2′-(thymine-1-yl)ethyluridine (**11**) in 37% yield. When the same procedure was repeated with *N*^3^-benzoyluridine (**10**) the double-headed nucleoside **11** was obtained in 67% yield (Scheme 3) [35].

The double-headed nucleoside **11** was dimethoxytritylated and phosphitylated following the standard procedures and incorporated once into a 13-mer oligodeoxynucleotide and an LNA-modified oligodeoxynucleotide sequence, and four-times in the middle of a 12-mer oligodeoxynucleotide sequence in order to study the effect of the additional nucleobase in duplexes, bulged duplexes, and in three way junctions [35]. The designed double-headed nucleoside was found to be reasonably well tolerated in duplexes and stabilized three-way junctions. Significant conformational changes in these secondary structures have also been induced [35].

**Scheme 4:**
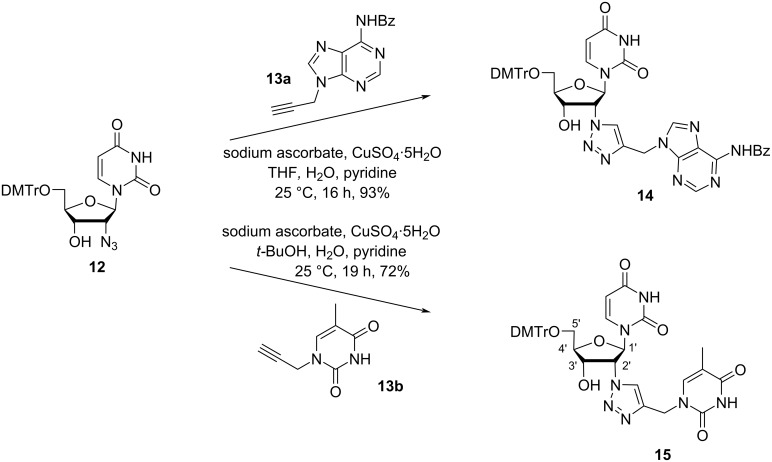
Double-headed nucleosides **14** and **15** obtained by click reaction.

Nielsen and co-workers [43] synthesized 2′-(4-(thymin-1-ylmethyl)-1,2,3-triazole-1-yl)- and 2′-(4-(*N*^6^-benzoyladenine-9-ylmethyl)-1,2,3-triazole-1-yl)-substituted double-headed nucleosides of 2′-deoxy-5′-*O*-(4,4′-dimethoxytrityl)uridine (**14** and **15**) from the nucleoside azide **12** which in turn was obtained by the nucleophilic opening of *O-*2,2′-anhydrouridine [44]. The azido nucleoside **12** was reacted with *N*^6^-benzoyl-*N*^9^-propargyladenine (**13a**) and *N*^1^-propargylthymine (**13b**) via a CuAAC reaction where the triazole-containing linker connected the additional thymine or adenine to the 2′-position of 2′-deoxyuridine forming the double-headed nucleosides **14** and **15**, respectively (Scheme 4) [43].

Both double-headed nucleosides **14** and **15**, when incorporated into oligonucleotides were found to stabilize three-way junction in both DNA–DNA and DNA–RNA duplexes and when introduced into a (+1)-zipper motif, cross strand interactions were observed in a DNA–DNA duplex [43].

**Scheme 5:**
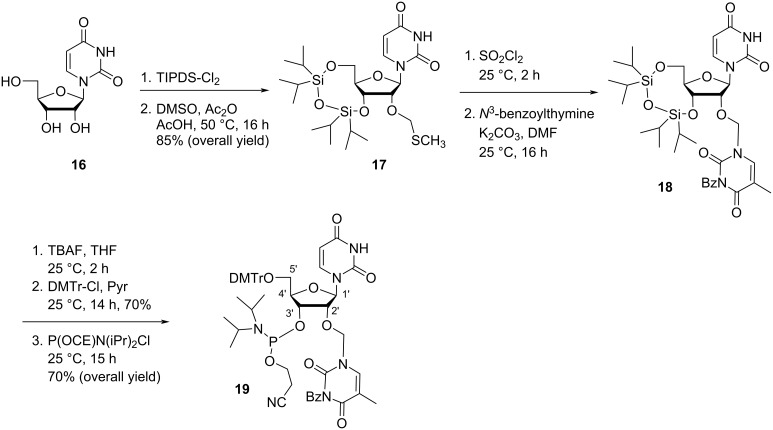
Synthesis of the double-headed nucleoside **19**.

TIPDS protection of uridine (**16**), followed by the treatment of the product with acetic anhydride/acetic acid in DMSO produced the protected nucleoside **17** [45–46] (Scheme 5). Next, the fully protected nucleoside **17** was subjected to chlorination using thionyl chloride in dichloromethane, followed by the treatment of the product with *N*^3^-benzoylthymine under basic conditions (K_2_CO_3_ in DMF) to produce the nucleoside **18**. The removal of the *tert*-butyldimethylsilyl group from nucleoside **18**, followed by dimethoxytritylation at the primary hydroxy and phosphitylation at the secondary hydroxy group afforded the double-headed nucleoside monomer **19** (Scheme 5) [45].

The synthesized double-headed nucleoside **19** was introduced in oligonucleotides and its impact on the secondary nucleic acid structure was studied. It was revealed that the double-headed nucleoside **19** was well accommodated in a hybrid DNA:RNA duplex and stabilized bulged duplex and three way junctions [45]. The potential of the double-headed nucleoside **19** in secondary nucleic acid structures was compared with the earlier reported monomer **11** and found to be inferior to double-headed nucleoside **11** due to the 3′-endo conformation which placed the 2′-substituent towards the minor groove rather than to the duplex core [35].

**Scheme 6:**
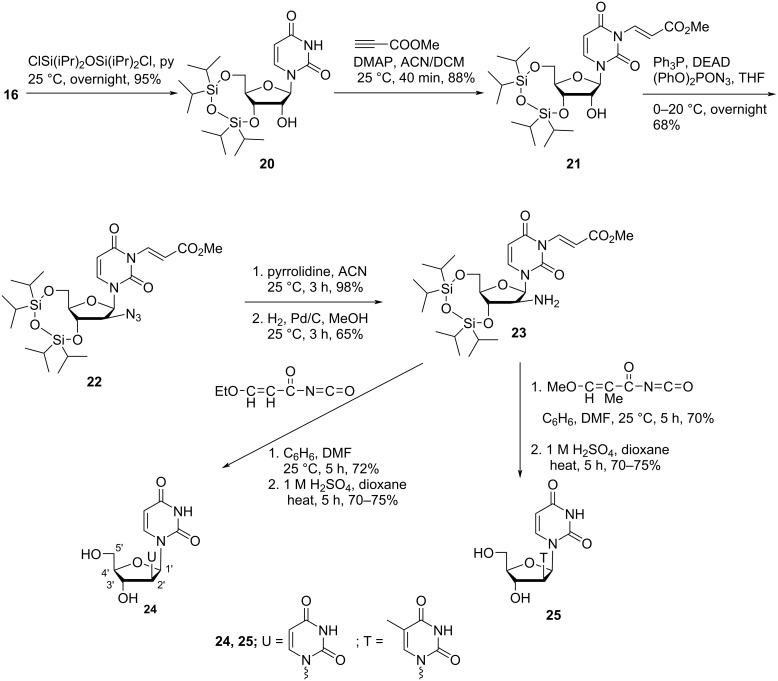
Synthesis of the double-headed nucleosides **24** and **25**.

Vilarrasa and co-workers [47] synthesized 2′-uracil-1-yl and 2′-thymin-1-yl derivatives of 2′-deoxythymidine starting from uridine (**16**). The synthesis started with the TIPDS protection of **16** followed by introduction of an azide group in the C-2′ position of the molecule to afford nucleoside **22**. The treatment of azide **22** with pyrrolidine in acetonitrile followed by hydrogenation afforded aminonucleoside **23**, which was used as a key intermediate for the synthesis of the double-headed nucleosides **24** and **25** (Scheme 6) [47].

**Scheme 7:**
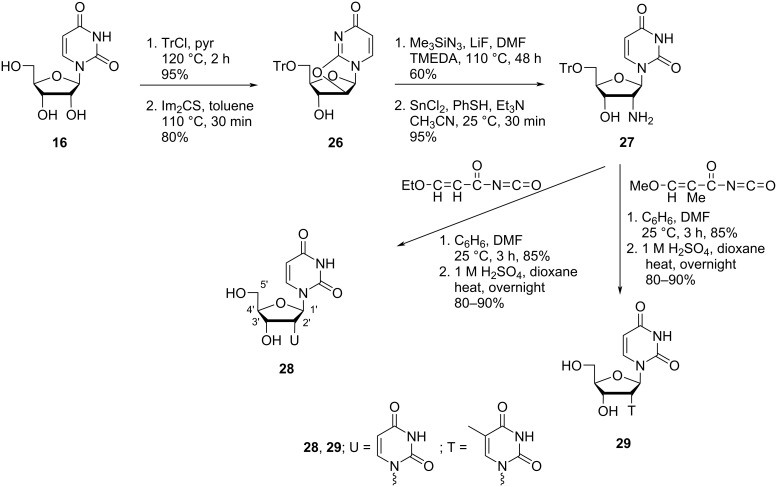
Synthesis of double-headed nucleosides **28** and **29**.

The same group [47] also synthesized the C-2′ isomeric nucleosides **28** and **29**, i.e., with inverted configuration at C-2′ as compared to nucleosides **24** and **25** (Scheme 7). The synthesis of these two nucleosides was carried out through the formation of the anhydro nucleoside **26** and its transformation into the aminonucleoside **27**. The key intermediate nucleoside **27** was then treated with 3-ethoxypropenoyl isocyanate or 3-methoxy-2-methylpropenoyl isocyanate in a mixture of benzene and DMF, followed by acidification with sulfuric acid affording the nucleosides **28** and **29**, respectively in high yields (Scheme 7) [47].

**Scheme 8:**
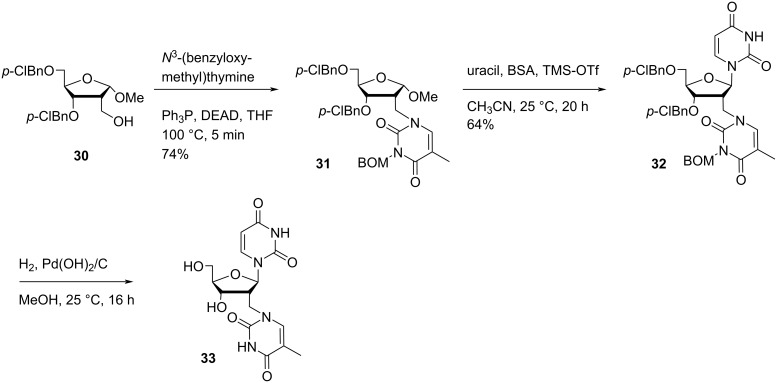
Synthesis of double-headed nucleoside **33**.

Nielsen and co-workers [33] synthesized the double-headed nucleoside 2′-*C*-(thymine-1-yl)methyl-2′-deoxyuridine (**33**) starting from the ribose derivative 3,5-bis-*O*-(*p*-chlorobenzyl)-2-deoxy-2-hydroxymethyl-α-ᴅ-ribofuranose (**30**) which in turn was synthesized from ᴅ-ribose in six steps following a procedure reported in the literature [48]. The Mitsunobu reaction of ribose derivative **30** and *N*^3^-(benzyloxymethyl)thymine afforded nucleoside **31** which was subjected to Vorbrüggen coupling with silylated uracil to give the protected double-headed nucleoside **32**. Global deprotection of **32** using palladium-catalyzed hydrogenation conditions resulted in the formation of the targeted double-headed nucleoside **33** (Scheme 8) [33].

The double-headed nucleoside **33** was dimethoxytritylated, phosphitylated, and incorporated into duplex and its ability to recognize complementary base pairs was monitored by UV melting curve analysis [33]. Hybridization data revealed that the synthesized double-headed nucleotide recognized itself either through formation of Watson–Crick base pairs with two complementary adenosines or through the formation of T:T (thymine:thymine) base pairs that resulted in the formation of two novel nucleic acid motifs. The novel nucleic acid motifs could be incorporated either single or multiple times in dsDNA duplexes without altering its stability. It was revealed by molecular dynamics (MD) simulations that the DNA sugar–phosphate backbone accommodated modified nucleotide by stretching or curling up as required and all the four base pairs based upon the structure of the synthesized double-headed nucleotide could be accommodated in the similar way as the T:A (thymine:adenine) base pair in the motif. The nucleic acid motifs may also be used in designing nanoscale DNA structures where a specific duplex twist is required [33].

**Scheme 9:**
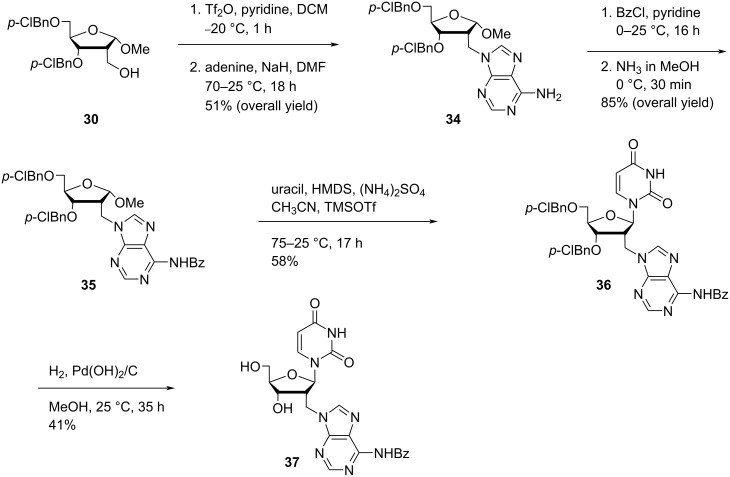
Synthesis of double-headed nucleoside **37**.

Nielsen and co-workers [34] also synthesized the double-headed nucleoside 2′-*C*-(*N*^6^-benzoyladenine-9-yl)methyl-2′-deoxyuridine (**37**) starting from ribose derivative 3,5-bis-*O*-(*p*-chlorobenzyl)-2-deoxy-2-hydroxymethyl-α-ᴅ-ribofuranose (**30**) [48]. The ribose derivative **30** was then reacted with triflic anhydride in the presence of pyridine followed by reaction with adenine in the presence of sodium hydride to afford nucleoside **34**. The nucleoside **34** was further reacted with benzoyl chloride to afford the fully protected nucleoside **35** which upon further reaction with silylated uracil in the presence of tin(IV) chloride via Vorbrüggen coupling afforded the protected double-headed nucleoside **36**. The nucleoside **36** was finally deprotected in the presence of Pd(OH)_2_/C under hydrogen atmosphere to generate the double-headed nucleoside **37** (Scheme 9) [34].

The double-headed nucleoside **37** was dimethoxytritylated, phosphitylated, and incorporated into 11- to 13-mer oligonucleotides using the standard automated phosphoramidite method. The UV-based melting temperature (*T*_m_) of hybrids of the modified oligonucleotides with complementary DNA strands were studied. The analysis of the melting temperature of the resulting duplex revealed that the synthesized double-headed nucleotide behaved as a compressed dinucleotide and combination of all natural nucleobases on compressed scaffold can form Watson–Crick base pairs with complementary bases [34].

**Scheme 10:**
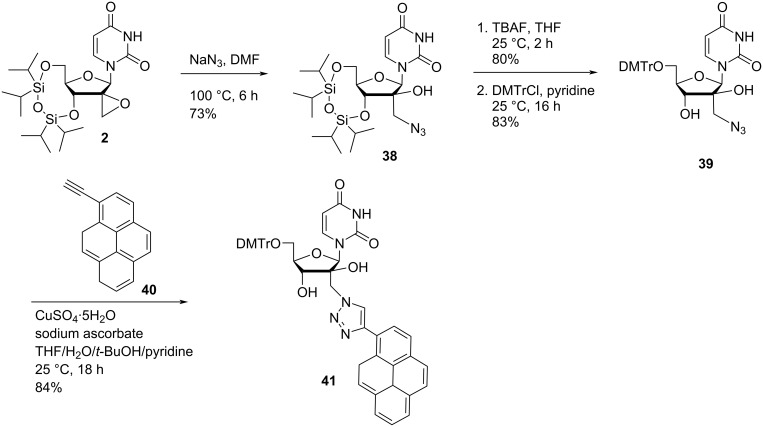
Synthesis of the double-headed nucleoside 1-(5′-*O*-(4,4′-dimethoxytrityl)-2′-*C*-((4-(pyren-1-yl)-1,2,3-triazole-1-yl)methyl)arabinofuranosyl)uracil (**41**).

Nielsen and co-workers [23] synthesized the double-headed nucleoside 1-(5′-*O*-(4,4′-dimethoxytrityl)-2′-*C*-((4-(pyren-1-yl)-1,2,3-triazole-1-yl)methyl)arabinofuranosyl)uracil (**41**) starting from spironucleoside **2** which in turn was synthesized from uridine following a procedure reported in the literature [30,32,49]. The spironucleoside **2** was then reacted with sodium azide to afford the *arabino*-uridine **38** with an azidomethyl group in the C-2′ position. The *arabino*-uridine **38** was reacted with TBAF and 4,4′-dimethoxytrityl chloride to afford nucleoside **39** which was reacted with 1-ethynylpyrene (**40**) under copper-catalyzed alkyne–azide cycloaddition (CuAAC) reaction conditions to yield the double-headed nucleoside **41** (Scheme 10) [23].

The double-headed nucleoside **41** was phosphitylated and then incorporated into oligonucleotides and was found to form highly stable DNA duplexes and three way junctions. There was a four-fold increase in the intensity of the pyrene excimer signal observed when an oligonucleotide containing two incorporations of the double-headed nucleoside **41** hybridized with an RNA target whereas the pyrene–pyrene excimer band almost vanished when the oligonucleotide was hybridized with a DNA target. The double-headed nucleoside **41** has potential in DNA invader probes as well as in RNA targeting and detection [23].

#### 1,3-Furanosyl double-headed nucleosides

In this section, all double-headed nucleosides with furanosyl ring structures are collected. The first nucleobase is attached at the anomeric position of the furanosyl ring structure and the second nucleobase is connected to the C-3′ position with or without a linker (Figure 1).

**Scheme 11:**
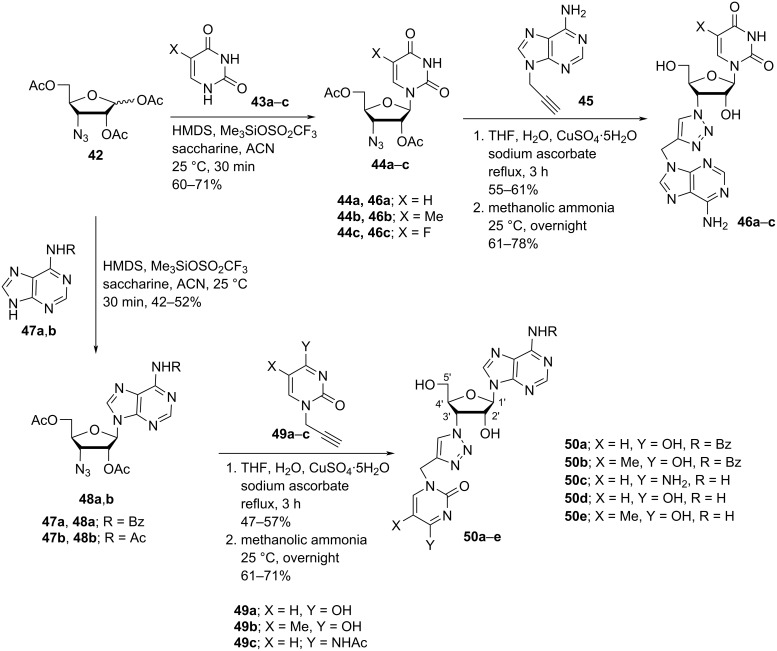
Synthesis of triazole-containing double-headed ribonucleosides **46a–c** and **50a–e**.

Leonaidas and co-workers [36] have synthesized 3′-(4-((adenine-9-yl)methyl)-1,2,3-triazol-1-yl)-substituted double-headed nucleosides of 1-(β-ᴅ-ribofuranosyl)uracil/thymine/5-fluorouracil **46a–c** and 3′-(4-((pyrimidin-1-yl)methyl)-1,2,3-triazol-1-yl)-substituted double-headed nucleosides of 9-(β-ᴅ-ribofuranosyl)adenine/*N*^6^-benzoyladenine **50a–e**. The synthesis started from C-3-azidoribofuranose **42** which in turn was obtained from 1,2:5,6-di-*O*-isopropylidene-ᴅ-glucose [50]. Furanoside **42** was reacted with the silyl-protected nucleobases **43a–c** and **47a**,**b** in the presence of trimethylsilyl trifluoromethanesulfonate in acetonitrile to give the 3′-azido-3′-deoxy-β-ᴅ-ribonucleosides **44a–c** and **48a**,**b** via Vorbrüggen coupling reaction. The nucleosides were further reacted with propargylated nucleobases through a copper-catalyzed azide–alkyne cycloaddition (CuAAC) reaction followed by treatment with methanolic ammonia to give the C-3′-substituted double-headed ribofuranonucleosides **46a–c** and **50a–e** (Scheme 11) [36].

The double-headed nucleosides **46a–c** and **50a–e** were evaluated for their inhibitory potency towards RNase A and eosinophil-derived neurotoxin (EDN). Among all the nucleosides, the double-headed nucleoside **50c** showed a stronger preference for EDN than for ribonuclease A whereas all other derivatives were found to be more specific for ribonuclease A [36].

**Scheme 12:**
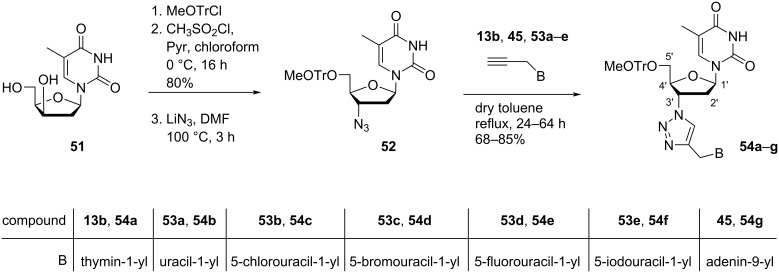
Synthesis of double-headed nucleosides **54a–g**.

Lazrek et al. [51] synthesized C-3'-modified double-headed nucleosides **54a–g** where a 1,2,3-triazol ring acts as linker of the nucleobase and the sugar moiety. First, seven *N*^9^/*N*^1^-propargylpurine/pyrimidine nucleobases **13b**, **45**, and **53a–g** were synthesized by treating the nucleobases with propargyl bromide in the presence of K_2_CO_3_. The synthesis of compounds **54a–g** started with β-ᴅ-lyxofuranosylthymine (**51**), which was first methoxytritylated at the C-5′ primary hydroxy position followed by mesylation of the C-3′ secondary hydroxy position. The subsequent treatment with sodium azide in DMF afforded the corresponding nucleoside **52** [52]. Triazolylation of compound **52** with the nucleobases **13b**, **45**, and **53a–g** by refluxing the substrates in toluene afforded the targeted 5′-*O*-monomethoxytritylated nucleosides **54a–g** (Scheme 12) [51].

**Scheme 13:**
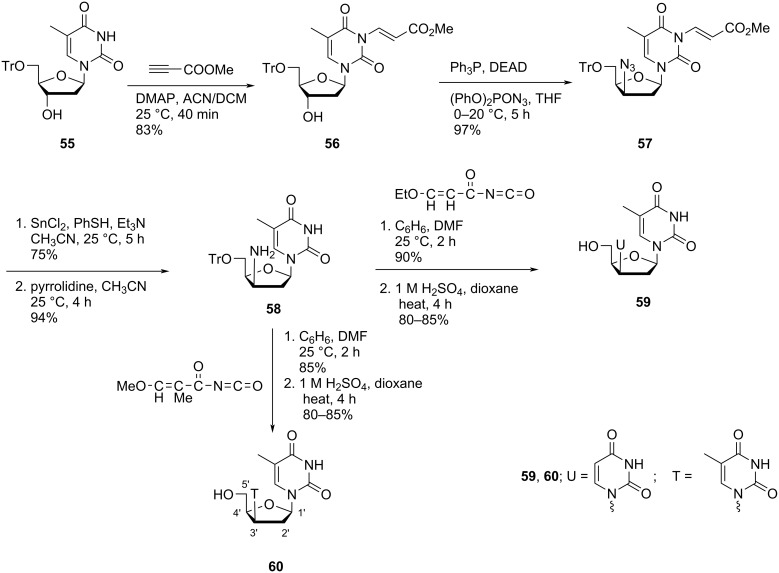
Synthesis of double-headed nucleosides **59** and **60**.

Vilarrasa and co-workers [47] synthesized 3′-uracil-1-yl and 3′-thymin-1-yl derivatives of 2′-deoxythymidine, i.e., compounds **59** and **60** starting from 5′-*O*-tritylthymidine (**55**). The tritylated thymidine **55** first was converted to the protected azide derivative **57** in two steps, followed by its reduction in the presence of tin(II) chloride, thiophenol and triethylamine and treatment with pyrrolidine in acetonitrile to afford the C-3’-aminonucleoside **58**. The reaction of this key intermediate with 3-ethoxypropenoyl isocyanate or 3-methoxy-2-methylpropenoyl isocyanate in a solvent mixture of benzene and DMF, followed by acidification with sulfuric acid produced the desired nucleosides **59** and **60**, respectively (Scheme 13) [47].

**Scheme 14:**
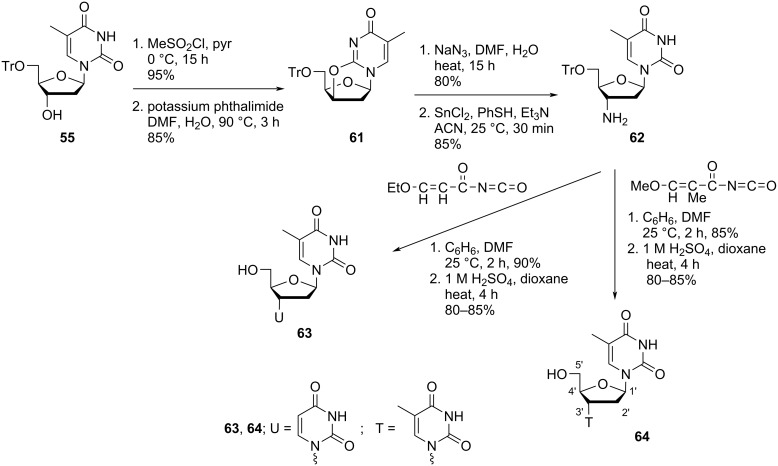
Synthesis of the double-headed nucleosides **63** and **64**.

Vilarrasa and co-workers [47] also synthesized the double-headed nucleosides **63** and **64** with downwards orientation of the additional nucleosides at the C-3′ position. The synthesis was carried out via formation of anhydride **61**. Azidation, followed by reduction of the corresponding nucleoside with tin chloride produced nucleoside **62** which was treated as a key intermediate for the production of the double-headed nucleosides **63** and **64**. Reaction of nucleoside **62** with 3-ethoxypropenoyl isocyanate or 3-methoxy-2-methylpropenoyl isocyanate in a solution mixture of benzene and DMF, followed by acidification with sulfuric acid produced nucleosides **63** and **64**, respectively (Scheme 14) [47].

#### 1,4-Furanosyl double-headed nucleosides

A literature search revealed two different categories of 1,4-furanosyl double-headed nucleosides. In the first category, the first nucleobase was a natural (attached at C-1′ position) and the second nucleobase was an aromatic moiety, which was attached at the C-4′ position without any linker (Figure 1). Whereas the second category of nucleosides contained first natural nucleobase at the C-1′ position and a second natural nucleobase attached at the C-4′ position with a methylene linker. The nucleosides of the second type may also contain a hydroxymethyl group at the C-4′ position.

**Scheme 15:**
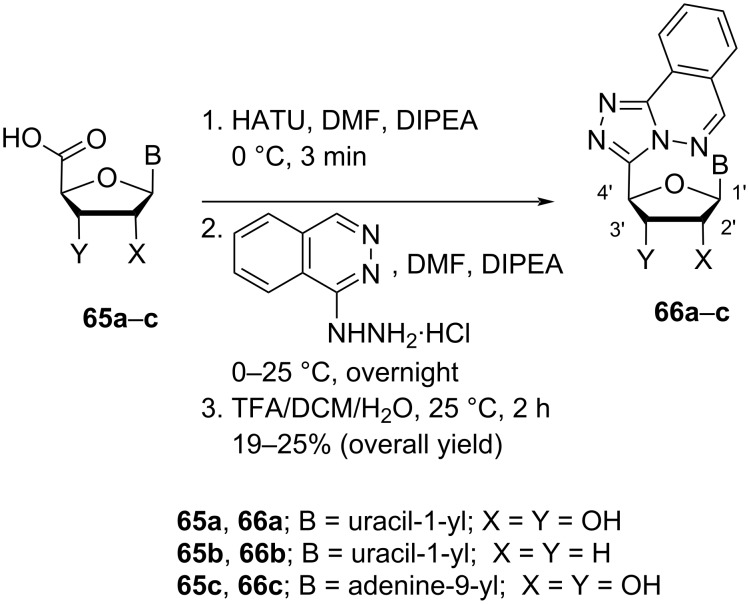
Synthesis of double-headed nucleosides **66a–c**.

Torrence and co-workers [11] synthesized triazolophthalazine-substituted double-headed nucleosides **66a–c** from uridine/adenosine-5′-carboxylic acids **65a–c** which in turn were prepared through the (2,2,6,6-tetramethylpiperidin-1-oxyl) (TEMPO) and 1,1-bis(acetoxy)iodobenzene (BAIB)-assisted oxidation of the 5′-hydroxymethylene group in adenosine/uridine by following the methodology developed by Epp and Widlanski [53]. The nucleoside-5′-carboxylic acids **65a–c** were reacted with 2-(1*H*-7-azabenzotriazol-1-yl)-1,1,3,3-tetramethyluronium hexafluorophosphate (HATU) as coupling reagent followed by reaction with phthalazin-1-ylhydrazin hydrochloride in DMF in the presence of diisopropylethylamine (DIPEA) as base to afford the double-headed nucleosides **66a–c** (Scheme 15) [11].

**Scheme 16:**
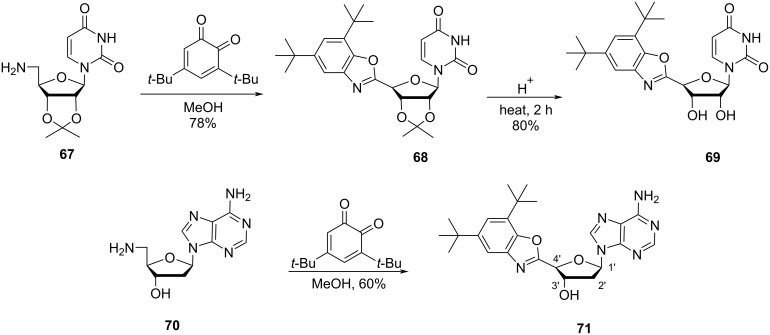
Synthesis of benzoxazole-containing double-headed nucleosides **69** and **71** from 5′-amino-5′-deoxynucleoside **67**.

Timoshchuk and Hogrefe [12] have synthesized the double-headed nucleosides (*R*)-*N*^1^-(4-(4,6-di-*tert*-butylbenzoxazol-2-yl)-2′,3′-*O*-isopropylidene-β-ᴅ-erythrofuranosyl)uracil (**69**) and (*R*)-*N*^9^-(4-(4,6-di-*tert*-butylbenzoxazol-2-yl)-2′-deoxy-β-ᴅ-erythrofuranosyl)adenine (**71**) by the reaction of 3,5-di-*tert*-butyl-1,2-benzoquinone with 5′-amino-5′-deoxy-2′,3′-*O*-isopropylideneuridine (**67**) and 5′-amino-2′,5′-dideoxyadenosine (**70**). The unprotected double-headed nucleoside (*R*)-*N*^1^-(4-(4,6-di-*tert*-butylbenzoxazol-2-yl)-β-ᴅ-erythrofuranosyl)uracil (**69**) was obtained by acidic hydrolysis of the intermediate benzoxazole derivative **68** (Scheme 16) [12].

**Scheme 17:**
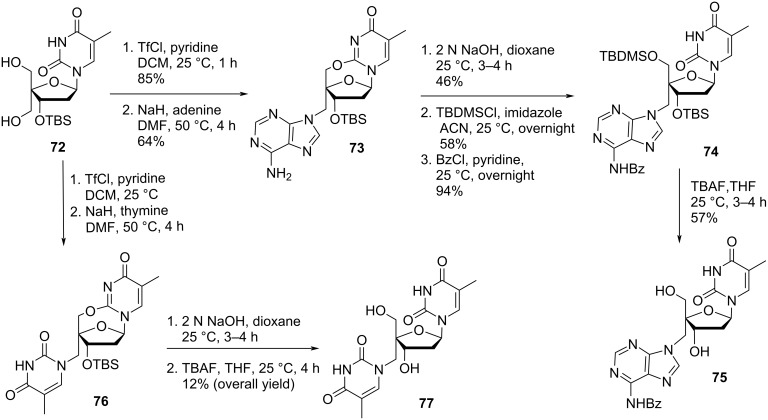
Synthesis of 4′-*C*-((*N*^6^-benzoyladenin-9-yl)methyl)thymidine (**75**) and 4′-*C*-((thymin-1-yl)methyl)thymidine (**77**).

Herdewijn and co-workers [54] synthesized the double-headed nucleoside monomers 4′-*C*-((*N*^6^-benzoyladenin-9-yl)methyl)thymidine (**75**) and 4′-*C*-((thymin-1-yl)methyl)thymidine (**77**) starting from 3′-*O*-(*tert*-butyldimethylsilyl)-4′-(hydroxymethyl)thymidine (**72**) which was conveniently synthesized from thymidine in five steps as reported in the literature [54–56]. The nucleoside **72** was then converted into the corresponding triflate derivative which was further reacted with the nucleobases adenine or thymine to afford compounds **73** and **76**, respectively (Scheme 17) [54].

The *tert*-butyldimethylsilyl-protected (TBDMS) nucleoside **76** was first hydrolyzed using NaOH, which was followed by TBDMS deprotection using tetra-*n*-butylammonium fluoride (TBAF) in tetrahydrofuran (THF) to afford the double-headed nucleoside **77**. The TBDMS-protected nucleoside **73** was first hydrolyzed using NaOH followed by the reaction with TBDMSCl and benzoyl chloride to get the *N*^6^-benzoyl-3’,5’-*O*-diTBDMS-protected nucleoside **74**. Removal of the silyl-protecting groups in the double-headed nucleoside **74** with TBAF in THF resulted in the formation of the desired doubled-headed nucleoside **75** (Scheme 17) [54].

The double-headed nucleosides **75** and **77** were 4-methoxytritylated and phosphitylated following the standard procedures and incorporated into oligonucleotides. Extrahelical A-T base interactions were observed when these double-headed nucleoside monomers were placed in opposite strands of the duplex with separation of one regular base pair from each other [54].

#### 1,5-Furanosyl double-headed nucleosides

In this category, the nucleosides contain the first natural nucleobase at the C-1′ position and the second natural nucleobase/aromatic moiety/heterocyclic ring attached at the C-5′ position with or without a linker (Figure 1).

**Scheme 18:**
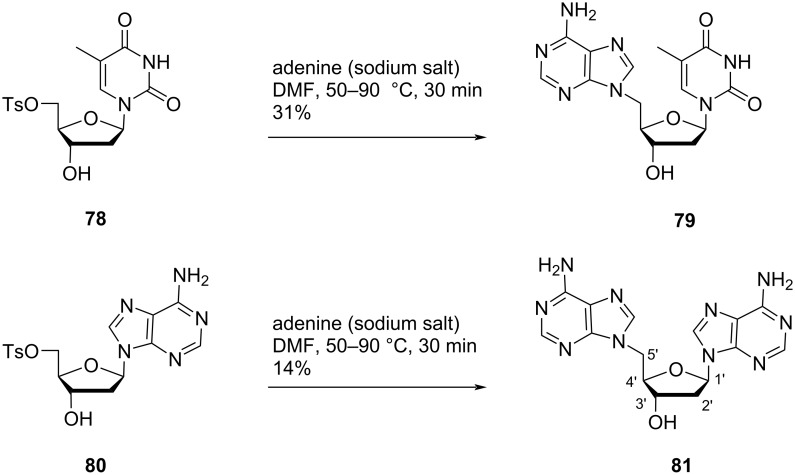
Synthesis of double-headed nucleosides 5′-(adenine-9-yl)-5′-deoxythymidine (**79**) and 5′-(adenine-9-yl)-2′,5′-dideoxyadenosine (**81**).

Shen and co-workers [57–58] proposed the synthesis of the double-headed nucleosides 5′-(adenine-9-yl)-5′-deoxythymidine (**79**) and 5′-(adenine-9-yl)-2′,5′-dideoxyadenosine (**81**) from 2′-deoxy-5′-*O*-tosylthymidine/adenosine **78** and **80**, respectively. The 2′-deoxy-5′-*O*-tosyl nucleosides were reacted with the sodium salt of adenine in DMF to afford the double-headed nucleosides **79** and **81**, respectively (Scheme 18) [57–58].

**Scheme 19:**
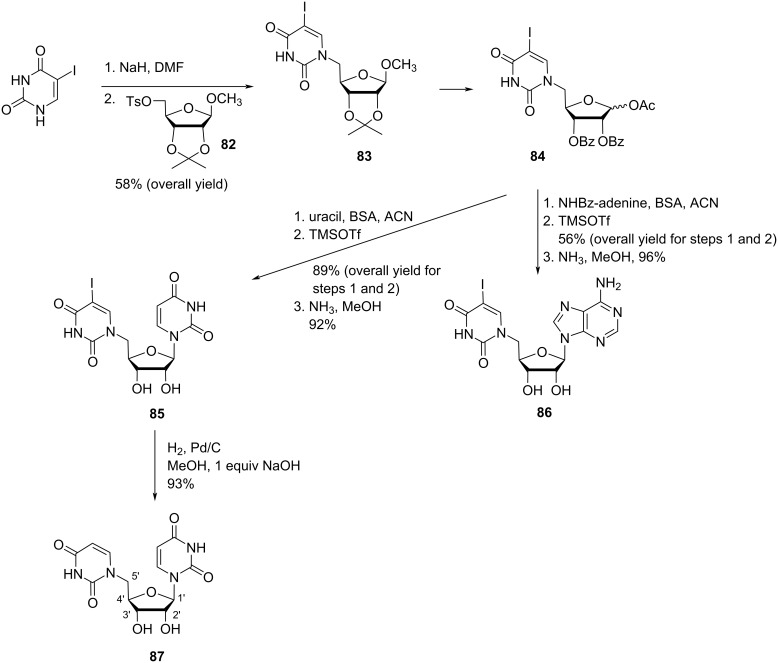
Synthesis of double-headed nucleosides **85–87** via reversed nucleosides methodology.

Žinić and co-workers [59] synthesized 5′-(5-iodouracil-1-yl)uridine (**85**), 5′-(5-iodouracil-1-yl)-5′-deoxyadenosine (**86**) and 5′-(uracil-1-yl)-5′-deoxyuridine (**87**) starting from the “reversed” 5-iodouracil-1-yl nucleoside **83** which in turn was synthesized by reacting the sodium salt of 5-iodouracil with isopropylidene-protected ribofuranoside **82** [55,59]. The reversed nucleoside **83** was next suitably protected to form nucleoside **84** which was then reacted with either uracil or *N*^6^-benzoyladenine via Vorbrüggen’s method of nucleobase coupling to produce the double-headed nucleosides **85** and **86**. Catalytic hydrogenolysis of the iodinated double-headed nucleoside **85** gave the nucleoside **87** (Scheme 19) [59].

**Scheme 20:**
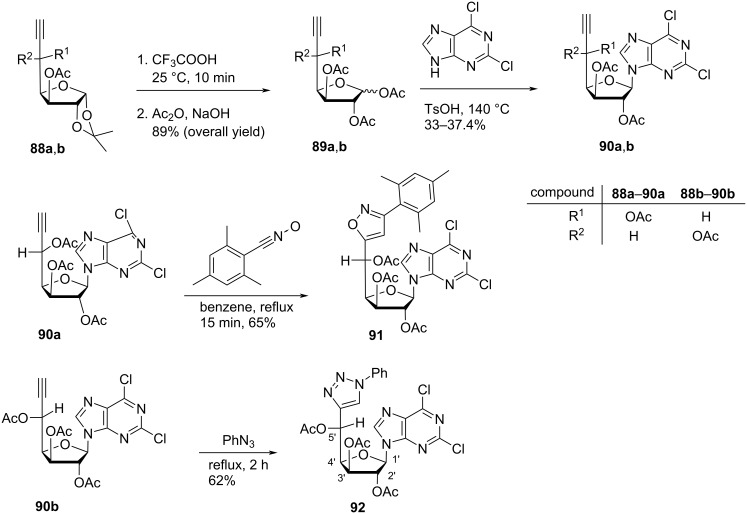
Double-headed nucleosides **91** and **92** derived from ω-terminal-acetylenic sugar derivatives **90a,b**.

Horton and Tsai [13] synthesized double-headed nucleosides 2,6-dichloro-9-(2,3,5-tri-*O*-acetyl-5-*C*-(3-mesitylisoxazol-5-yl)-α-ʟ-idopentofuranosyl)-9*H*-purine (**91**) and 2,6-dichloro-9-(2,3,5-tri-*O*-acetyl-5-*C*-(1-phenyl-1,2,3-triazol-4-yl)-β-ᴅ-glucopentofuranosyl)-9*H*-purine (**92**) starting from 3,5-di-*O*-acetyl-6,7-dideoxy-1,2-*O*-isopropylidene-ʟ-ido/α-ᴅ-gluco-hept-6-ynofuranoses **88a**,**b** The ʟ-ido- and ᴅ-gluco precursors **88a,b** were reacted with trifluoroacetic acid followed by acetic anhydride to afford the 1,2,3,5-tetra-*O*-acetyl nucleoside analogs **89a** and **89b**, respectively. Montgomery and Hewson base coupling reaction [60] of 1,2,3,5-tetra-*O*-acetyl nucleoside analogues **89a** and **89b** with 2,6-dichloropurine under acidic conditions resulted in the formation of mononucleoside analogs **90a,b**. The nucleoside **90a** was reacted with mesitylnitrile to give the double-headed nucleoside **91**, whereas nucleoside **90b** was reacted with phenylazide to give the double-headed nucleoside **92** (Scheme 20) [13].

**Scheme 21:**
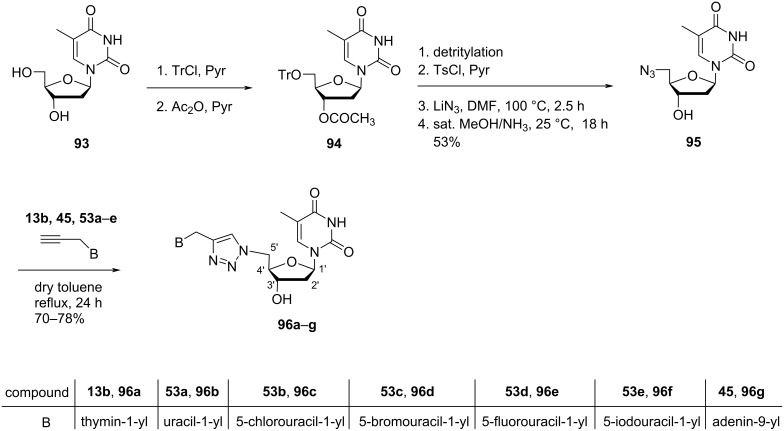
Synthesis of double-headed nucleosides **96a–g**.

Lazrek et al. [51] synthesized C-5′-modified double-headed nucleosides **96a–g**, where a 1,2,3-triazolo ring acted as the linker between the nucleobase and the sugar moiety. First, seven *N*^9^/*N*^1^-propargylpurine/pyrimidine nucleobases **13b, 45** and **53a–e** were synthesized by treating the nucleobases with propargyl bromide in the presence of K_2_CO_3_. Nucleoside **94** was synthesized from thymidine (**93**) which was first tritylated at the C-5′ primary hydroxy position followed by acetylation at the C-3′ secondary hydroxy group [61]. Next, detritylation and tosylation of the protected nucleoside **94** followed by treatment with lithium azide in DMF and saturated methanolic ammonia solution afforded nucleoside **95**. Refluxing of nucleoside **95** with **13b, 45** and **53a–e** in toluene produced the desired nucleosides **96a–g** (Scheme 21) [51].

**Scheme 22:**
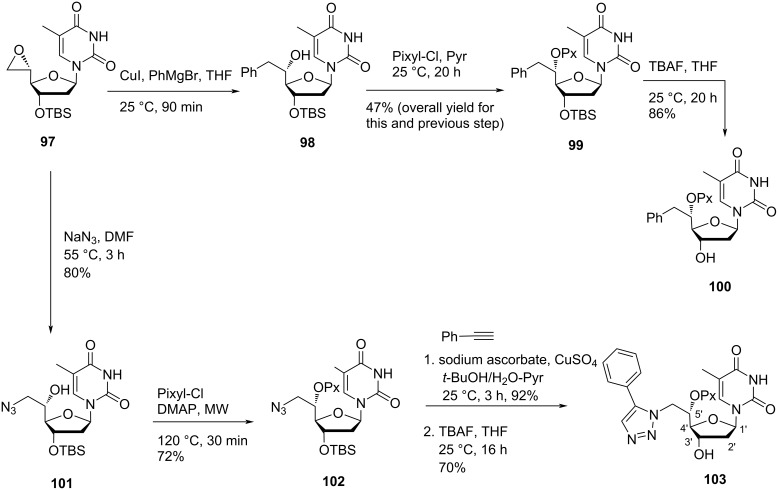
Synthesis of double-headed nucleosides **100** and **103**.

Shaikh et al*.* [14] reported the synthesis of double-headed nucleosides where an aromatic moiety or a nucleobase is attached at the C-5′ position of the nucleoside. The synthetic methodology started with the 5′-epoxide **97**, which was synthesized from 3′-*O*-(*tert*-butyldimethylsilyl)thymidine in three steps, where the oxidation of the C-5′-hydroxy group followed by a Wittig reaction with methylenetriphenylphosphorane (Ph_3_P=CH_2_) produced the 5′-methylene derivative [62]. Finally, oxidation with *meta*-chloroperbenzoic acid (mCPBA) afforded the nucleoside **97**. Treatment of the nucleoside **97** with Grignard reagent PhMgBr in THF produced nucleoside **98**, whose secondary hydroxy group was protected by reaction with pixyl chloride to afford the nucleoside **99**. The removal of the *tert*-butyldimethylsilyl protecting group under standard conditions afforded the double-headed nucleoside **100** (Scheme 22) [14].

**Scheme 23:**
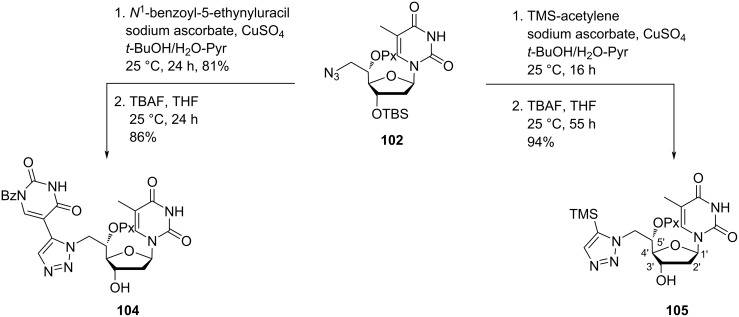
Double-headed nucleosides **104** and **105** with a triazole motif.

Opening of the epoxide ring in nucleoside **97** with sodium azide in DMF produced nucleoside **101**, whose secondary hydroxy group was protected by reaction with pixyl chloride to afford nucleoside **102**. The azido nucleoside **102** was a key intermediate, which was used for the synthesis of a variety of 1,2,3-triazolyl-linked double-headed nucleosides. Thus the treatment of azido nucleoside **102** with phenylacetylene in the presence of sodium ascorbate and copper sulfate in a solvent mixture of *t*-BuOH, water and pyridine, followed by the removal of the *tert*-butyldimethylsilyl protecting group gave nucleoside **103** (Scheme 22) [14]. Under similar reaction conditions, the treatment of nucleoside **102** with *N*^1^-benzoyl-5-ethynyluracil followed by desilylation produced the double-headed nucleoside **104**, whereas the reaction of the azido nucleoside **102** with trimethylsilylacetylene (TMS-acetylene) followed by desilylation produced the nucleoside **105** (Scheme 23) [14].

**Scheme 24:**
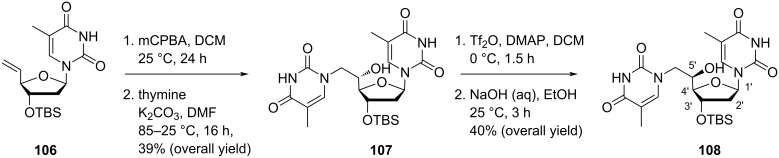
Synthesis of the double-headed nucleosides **107** and **108**.

Christensen et al. [31] synthesized the double-headed nucleoside 5′-(*S*)-*C*-(thymin-1-ylmethyl)-3′-*O*-(*tert*-butyldimethylsilyl)thymidine (**107**) by treating the 5′-olefinic nucleoside **106** with mCPBA in dichloromethane followed by reaction of the resulted product with thymine in the presence of K_2_CO_3_ in hot DMF. The targeted double-headed nucleoside 5′-(*R*)-*C*-(thymin-1-ylmethyl)-3′-*O*-(*tert*-butyldimethylsilyl)thymidine (**108**) was synthesized by treating the double-headed nucleoside **107** with triflic anhydride followed by basic hydrolysis (Scheme 24) [31].

Subsequently, the double-headed nucleoside **107** was incorporated into oligonucleotides [31,33,35,63–64] and when the duplex was generated with complementary DNA and RNA sequences, the additional nucleobase was positioned in the minor groove of the duplex. However, the presence of the additional nucleobase resulted in a thermal destabilization of the duplex as compared to unmodified duplexes. The introduction of two double-headed nucleosides in two complementary DNA sequences forming a DNA-zipper motif showed a stabilization of the duplex and increased base–base stacking interactions.

**Scheme 25:**
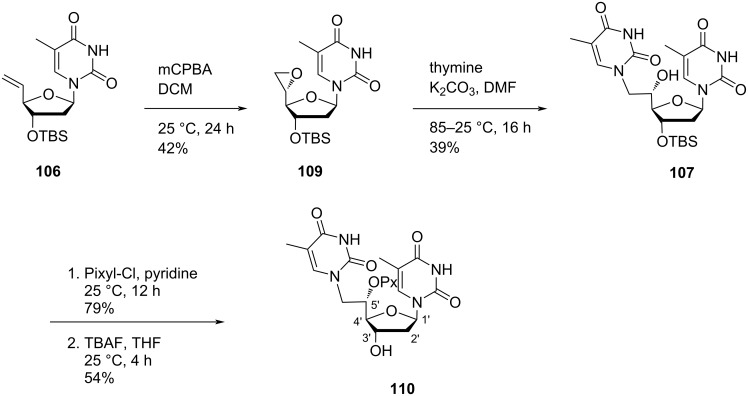
Synthesis of double-headed nucleoside **110** with additional nucleobase in 5′-(*S*)-*C*-position joined through methylene linker.

Nielsen and co-workers [30–31,65] synthesized double-headed nucleosides 5′-(*S*)-*C*-(thymine-1-yl/purin-9-yl)methyl-substituted double-headed nucleosides of thymidine **110–113** with additional nucleobase in the 5′(*S*)-C-position of thymidine. The double-headed nucleosides **110–113** were synthesized from the olefinic nucleoside **106**, which was converted into the epoxide **109** by treatment with mCPBA following the literature procedure [14,62]. The epoxide **109** so formed was reacted with thymine to afford nucleoside **107**, which on pixylation and removal of the *tert*-butyldimethylsilyl-protecting group in the presence of TBAF give double-headed nucleoside **110** (Scheme 25) [30–31,65].

**Scheme 26:**
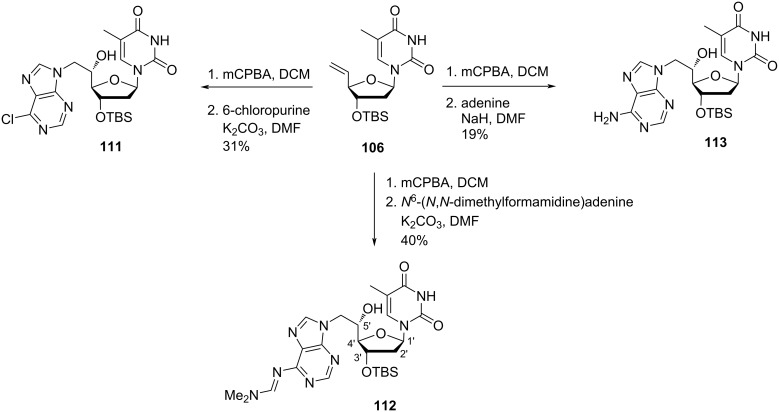
Synthesis of double-headed nucleosides **111–113** with additional nucleobases in the 5′-(*S*)-*C*-position joined through methylene linkers.

The epoxidation of the olefinic nucleoside **106** with mCPBA followed by reaction with 6-chloropurine, *N*^6^-(*N*,*N*-dimethylformamidine)adenine, or adenine in the presence of K_2_CO_3_/NaH in hot DMF afforded the double-headed nucleosides **111–113** (Scheme 26) [30–31,65].

These nucleoside monomers were converted into phosphoramidites and then incorporated into oligonucleotide sequences, followed by thermal hybridization studies that indicated that the 5′-(*S*)-C-position is ideal for placing an additional nucleobase in the minor groove and interstrand stacking effects decreased with an increase in the length of the linker [31,65].

**Scheme 27:**
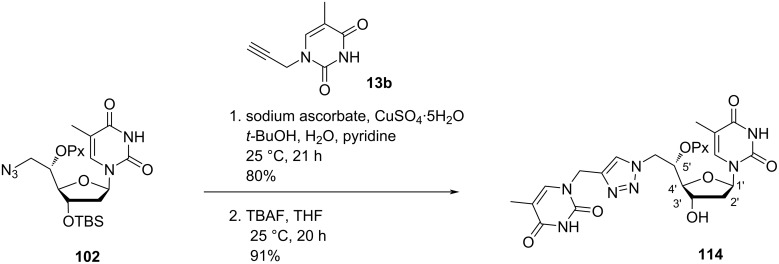
Synthesis of double-headed nucleoside **114** by click reaction.

Nielsen and co-workers [43] synthesized the double-headed nucleoside 5′-*O*-pixyl-5′(*S*)-*C*-(4-(thymin-1-yl-methyl)-1,2,3-triazol-1-yl)methylthymidine (**114**) from 3′-TBS-protected 5´- (*S*)-*C*-azidomethylthymidine **102** which was synthesized from 3′-*O*-TBS-protected thymidine [31,65–66]. The nucleoside azide **102** was then reacted with propargylated thymine via CuAAC reaction, and subsequent removal of TBS group in the presence of TBAF and THF afforded the double-headed nucleoside **114** where a triazole linker connected the additional thymine to the 5′-position of thymidine (Scheme 27) [43].

The incorporation of the double-headed nucleoside monomer **114** into oligonucleotides failed to stabilize three-way junctions [43] which is contrary to the double-headed nucleoside **11** which stabilized three-way junction very efficiently [35].

**Scheme 28:**
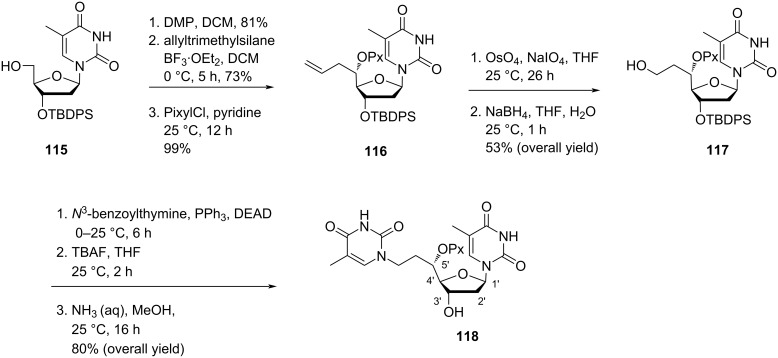
Synthesis of double-headed nucleosides **118** with an additional nucleobase at the 5′-(*S*)-C-position.

Nielsen and co-workers [30,65] synthesized the double-headed nucleoside 5′-(*S*)-*C*-(2-(thymine-1-yl)ethyl)thymidine (**118**) with an additional nucleobase at the 5′-(*S*)-*C*-position of thymidine. Double-headed nucleoside **118** was synthesized starting from 3′-*tert*-butyldiphenylsilyl (TBDPS)-protected thymidine **115** which was converted into the pixylated 5′(*S*)-*C*-allyl-substituted nucleoside **116** following previously reported procedures [65]. Then, the nucleoside **116** was converted into the primary alcohol **117** by treatment with OsO_4_ and oxidative cleavage by NaIO_4_ followed by reduction using NaBH_4_. The primary alcohol **117** was further converted into nucleoside monomer **118** by introduction of the second nucleobase thymine through Mitsunobu reaction followed by deprotection steps in the presence of TBAF and methanolic ammonia (Scheme 28) [30,65].

The nucleoside monomer **118** was phosphitylated and then incorporated into oligodeoxynucleotides but stabilization in the secondary structures due to additional thymine in combination with the ethylene linker in double-headed nucleoside **118** was not observed because of an increase in the length of the linker which is contrary to the double-headed nucleoside **110** which stabilized secondary structures very well due to shorter length of linker [30–31,65].

### Bicyclic double-headed nucleosides

In this section we have included the double-headed nucleoside monomers, which have a locked nucleic acid type conformation and the additional nucleobase is attached at one of the carbon or nitrogen atoms constituting the bridge (Figure 1). All examples discussed herewith are constituted by furanosyl carbohydrate moiety.

**Scheme 29:**
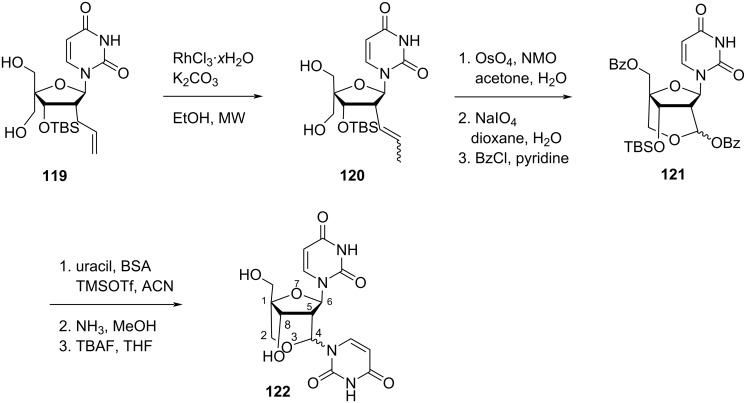
Synthesis of bicyclic double-headed nucleoside **122**.

Nielsen and co-workers [67] synthesized the bicyclic double-headed nucleoside (1*R*,4*R*/*S*,5*R*,6*R*,8*S*)-8-hydroxy-1-hydroxymethyl-4,6-di(uracil-1-yl)-3,7-dioxabicyclo[3.2.1]octane (**122**), where the additional nucleobase is attached at the bridge between C-2′ and C-4′. The synthesis of the aimed nucleoside **122** started from the nucleoside **119** which in turn was synthesized from uridine in six steps following a literature procedure [68]. The olefinic nucleoside **119** was subjected to a RhCl_3_-mediated allyl rearrangement to give nucleoside **120** as a mixture of *E*/*Z* isomers. Further, double bond cleavage of nucleoside **120** followed by benzoylation produced the benzoic acid ester of the hemiacetal analogue **121** which was finally converted into double-headed nucleoside **122** via Vorbrüggen coupling reaction followed by deprotection using methanolic ammonia and TBAF (Scheme 29) [67].

**Scheme 30:**
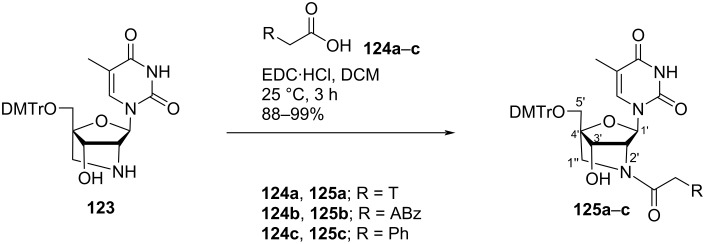
Synthesis of double-headed nucleosides **125a–c** derived from 2′-amino-LNA.

Madsen and co-workers [69] synthesized *N*^2^-(thymin-1-ylacetyl)-, *N*^2^-(*N*^6^-benzoyladenin-9-ylacetyl)-, and *N*^2^-phenylacetyl-substituted double-headed nucleosides of 1-(2′-amino-2′-deoxy-5-*O*-(4,4′-dimethoxytrityl)-2′-*N*,4′-*C*-methylene-β-ᴅ-ribofuranosyl)thymine **125a–c** by the reaction of 5′-*O*-dimethoxytritylated 2′-amino-LNA thymine nucleoside **123** with acetic acid derivatives, i.e., (thymin-1-yl)acetic acid (**124a**), (*N*^6^-benzoyladenin-9-yl)acetic acid (**124b**), and phenylacetic acid (**124c**) in the presence of EDC·HCl as condensation reagent (Scheme 30) [69].

The double-headed nucleoside monomers **125a–c** were incorporated into oligodeoxyribonucleotides via phosphoramidite derivatization of the C-3′ hydroxy group present in the moiety. The oligonucleotides thus synthesized were found to stabilize the duplex formed with complementary DNA [69].

**Scheme 31:**
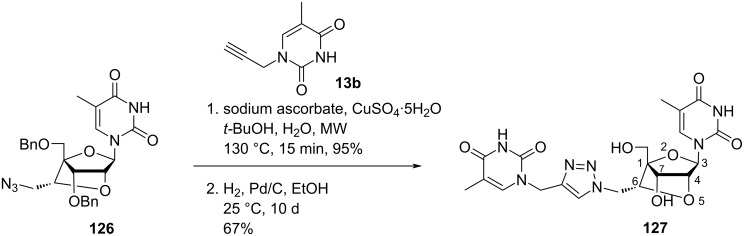
Double-headed nucleoside **127** obtained by click reaction.

Nielsen and co-workers [43,70] synthesized the double-headed nucleoside (1*S*,3*R*,4*R*,6*R*,7*S*)-7-hydroxy-1-(hydroxymethyl)-3-(thymin-1-yl)-6-(4-(thymin-1-ylmethyl)-1,2,3-triazol-1-yl)methyl-2,5-dioxabicyclo[2.2.1]heptane (**127**). The CuAAc reaction of nucleoside azide **126** with propargylated thymine followed by Pd-catalyzed debenzylation under hydrogen atmosphere resulted in the formation of the double-headed nucleoside **127**. In nucleoside **127** the triazole ring is attached to the additional thymine moiety via a methylene linker and connected to the 6′-position of an LNA-thymidine monomer, via another methylene linker (Scheme 31) [43,70]. Interestingly, the incorporation of the double-headed nucleoside **127** into oligonucleotides failed to stabilize three-way junctions [43].

### Base to base double-headed nucleosides

Base to base double-headed nucleosides contain an additional natural nucleobase/substituted or unsubstituted phenyl moiety/polyaromatic moiety/carbocyclic moiety/heterocyclic moiety attached at the C-5 position of the pyrimidine nucleobase (first nucleoside) with or without a linker. The sugar moieties associated with these nucleosides were either a 2′-deoxyribofuranosyl moiety or a bicyclic moiety (Figure 1).

**Scheme 32:**
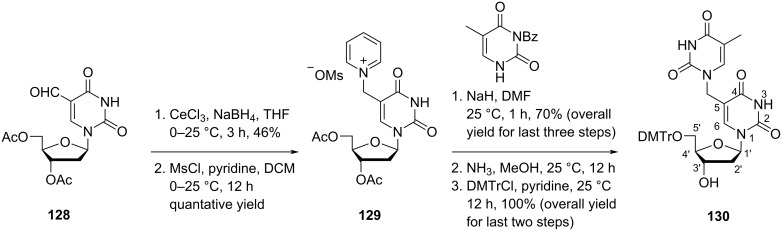
Synthesis of double-headed nucleoside **130**.

Nielsen and co-workers [71] synthesized the double-headed nucleoside 5′-*O*-(4,4′-dimethoxytrityl)-5-(thymin-1-yl)methyl-2′-deoxyuridine (**130**) with thymine attached to the C-5 position of 2′-deoxyuridine through a methylene linker. The double-headed nucleoside **130** was synthesized from 3′,5′*-O-*diacetyl-5-formyl-2′-deoxyuridine (**128**) which was reduced in the presence of NaBH_4_ followed by the treatment with MsCl in pyridine to get the nucleoside salt **129**. Next, the pyridinium group was replaced by an *N*^3^-protected thymine in basic medium followed by removal of the protecting groups and the selective DMTr protection of the C-5′-hydroxy group (Scheme 32) [71].

The double-headed nucleoside monomer 5′-*O*-(4,4′-dimethoxytrityl)-5-(thymin-1-yl)methyl-2′-deoxyuridine (**130**) was converted into the corresponding phosphoramidite at the C-3′-hydroxy group and then incorporated into oligonucleotides and was found to decrease the thermal stability of the duplexes [71].

**Scheme 33:**
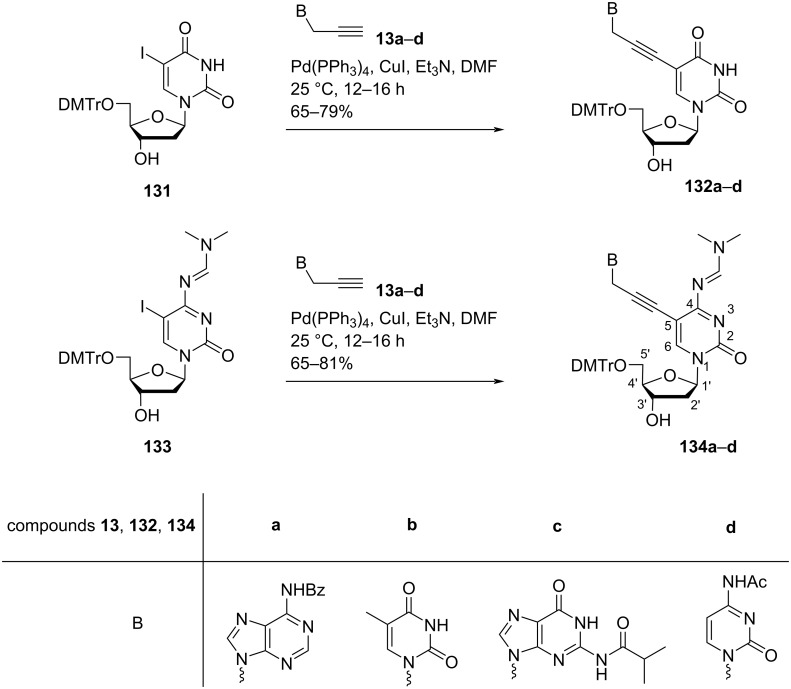
Double-headed nucleosides **132a–d** and **134a–d** synthesized by Sonogashira cross coupling reaction.

Nielsen and co-workers [72] synthesized 5-(3-(thymin-1-yl)propyn-1-yl)-, 5-(3-(*N*^4^-acetylcytosin-1-yl)propyn-1-yl)-, 5-(3-(*N*^6^-benzoyladenin-9-yl)propyn-1-yl), and 5-(3-(*N*^2^-isobutyrylguanin-9-yl)-substituted double-headed nucleosides of 5′-*O*-DMTr-protected 2′-deoxyuridine (**132a–d**) and 5′-*O*-DMTr-protected *N*^4^-(dimethylaminomethylene)-2′-deoxycytidine (**134a–d**) by using Sonogashira cross coupling reaction between the propargylated nucleobases, i.e*.*, 1-propargylthymine, *N*^4^-acetyl-1-propargylcytosine, *N*^6^-benzoyl-9-propargyladenine, and *N*^2^-isobutyryl-9-propargylguanine (**13a–d**) and 5′-*O*-DMTr-protected 5-iodo-2′-deoxyuridine (**131**) and *N*^4^-(dimethylaminomethylene)-2′-deoxycytidine (**133**) in 65–81% yield (Scheme 33).

The synthesized double-headed nucleosides were phosphitylated and incorporated into oligonucleotides and the melting temperatures were evaluated against unmodified DNA strands. Oligonucleotides with fourteen consecutive incorporations of different double-headed nucleosides were synthesized and the DNA duplexes showed increased stability owing to increased stacking interactions among the nucleobases of the opposite strands [72]. Molecular dynamics simulations demonstrated the exposure of Watson–Crick/Hoogsteen faces of additional nucleobases for their recognition in the major groove.

**Scheme 34:**
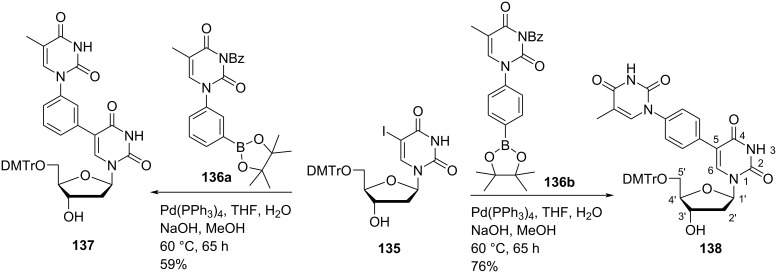
Synthesis of double-headed nucleosides **137** and **138** via Suzuki coupling.

Sharma and co-workers [73] synthesized 5-(3-(thymin-1-yl)phenyl)- and 5-(4-(thymin-1-yl)phenyl)-substituted double-headed nucleosides of 5′-*O*-dimethoxytrityl-2′-deoxyuridine (**137**, **138**) from 5′-*O*-DMTr-2′-deoxy-5-iodouridine (**135**). Boronic esters *N*^1^-(3-(4,4,5,5-tetramethyl-1,3,2-dioxaborolan-2-yl)phenyl) and *N*^1^-(4-(4,4,5,5-tetramethyl-1,3,2-dioxaborolan-2-yl)phenyl)-substituted *N*^3^-benzoylthymine (**136a,b**) were synthesized by *N*^3^-benzoylthymine from the procedure given by Gothelf and co-workers [74–75]. The boronic esters (**136a,b**) were coupled with 5′-*O*-DMTr-2′-deoxy-5-iodouridine (**135**) via Suzuki coupling to give double-headed nucleosides **137** and **138** (Scheme 34) [73].

**Scheme 35:**
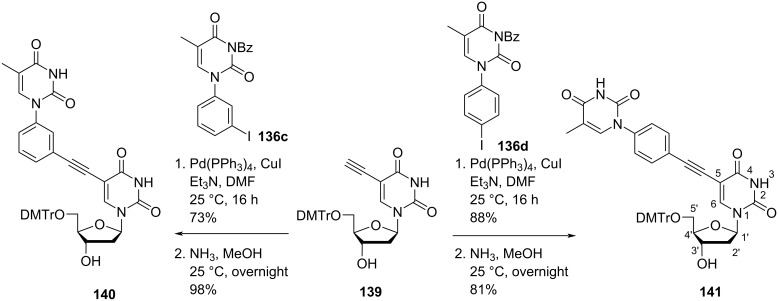
Synthesis of double-headed nucleosides **140** and **141** via Sonogashira cross coupling reaction.

The double-headed nucleosides 5′-*O*-dimethoxytrityl-5-(3-(thymin-1-yl)phenyl)ethynyl-2′-deoxyuridine (**140**) and 5′-*O*-dimethoxytrityl-5-(4-(thymin-1-yl)phenyl)ethynyl-2′-deoxyuridine (**141**) were synthesized via a Sonogashira cross coupling reaction between the *N*^1^-(3/4-iodophenyl)thymine derivatives **136c** and **136d** and 2′-deoxy-5-ethynyluridine derivative **139** (Scheme 35) [75].

All four nucleoside monomers were converted into phosphoramidites and then introduced into oligonucleotides. The thermal stability of DNA:DNA and DNA:RNA duplexes was determined and it was found that duplexes with a *meta*-substitution and a phenylacetylene linker were more stable than the corresponding *para*-substituted and phenyl-linker containing derivatives.

**Scheme 36:**
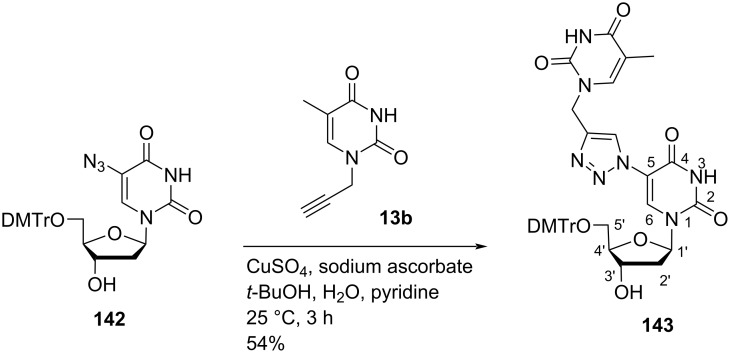
Synthesis of double-headed nucleoside **143**.

Nielsen and co-workers [71] synthesized the double-headed nucleoside 5′-*O*-(4,4′-dimethoxytrityl)-5-(4-(thymin-1-yl)methyl-1,2,3-triazol-1-yl)-2′-deoxyuridine (**143**) with an additional thymine attached to the 5-position of the 2′-deoxyuridine through a triazolomethylene linker. The double-headed nucleoside **143** was synthesized by the CuAAC reaction between 5-azido-5′-*O*-DMTr-2′-deoxyuridine (**142**) and 1-propargylthymine (**13b**) (Scheme 36) [71].

**Scheme 37:**
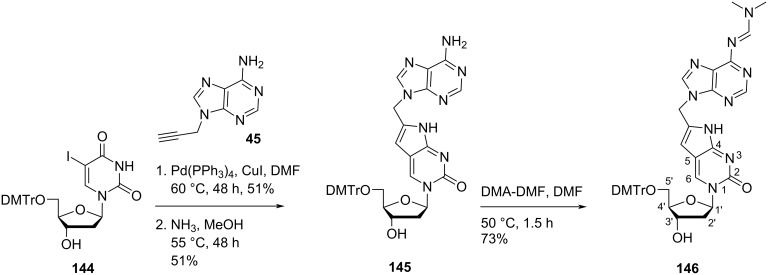
Synthesis of the double-headed nucleoside **146**.

Nielsen and co-workers [71] also synthesized the double-headed nucleoside 5′-*O*-(4,4′-dimethoxytrityl)-6-((*N*^6^-(dimethylaminomethylidenyl)adenin-9-yl)methyl-2′-deoxypyrrolocytidine (**146**) which has adenine attached to the 6-position of the pyrrolo-2′-deoxycytidine through a methylene linker. The double-headed nucleoside **146** was synthesized through the Sonogashira coupling reaction between 5′-*O*-DMTr-5-iodo-2′-deoxyuridine (**144**) and *N*^9^-propargyladenine (**45**) followed by treatment with methanolic ammonia and DMA–DMF (Scheme 37) [71].

Both double-headed nucleoside monomers **143** and **146** were phosphoramidated at the C-3′ hydroxy group and incorporated into oligonucleotides. The synthesized oligonucleotides were found to decrease the thermal stability of the duplexes. However, their potential in triplex forming oligonucleotides was also studied which concluded the formation of most stable triplexes with single incorporations of additional pyrimidine nucleobases connected via a propylene linker [71].

**Scheme 38:**
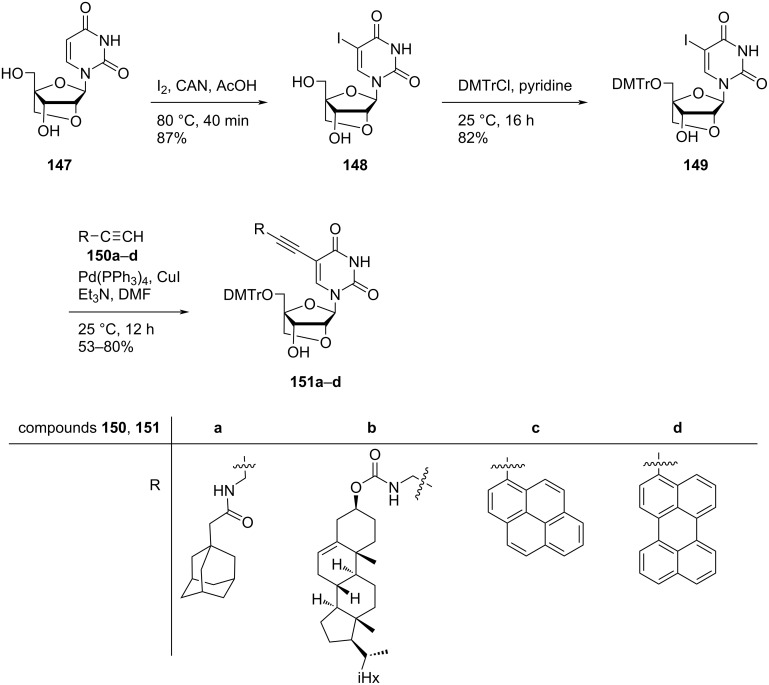
Synthesis of 5-*C*-alkynyl-functionalized double-headed nucleosides **151a–d**.

Hrdlicka and co-workers [24] synthesized 5-*C*-alkynyl-functionalized double-headed nucleosides **151a–d** starting from LNA uridine diol **147** which in turn was synthesized from diacetone-α-ᴅ-allose following a procedure reported in the literature [76]. LNA uridine diol **147** was reacted with iodine and ceric ammonium nitrate (CAN) in acetic acid to afford the nucleoside **148**. Nucleoside **148** was then 5′-*O*-dimethoxytritylated in the presence of DMTrCl (4,4′-dimethoxytrityl chloride) and pyridine. The 5′-*O*-dimethoxytritylated nucleoside **149** was further coupled with terminal alkynes **150a–d** under Sonogashira conditions to afford the double-headed nucleosides **151a–d** (Scheme 38) [24].

**Scheme 39:**
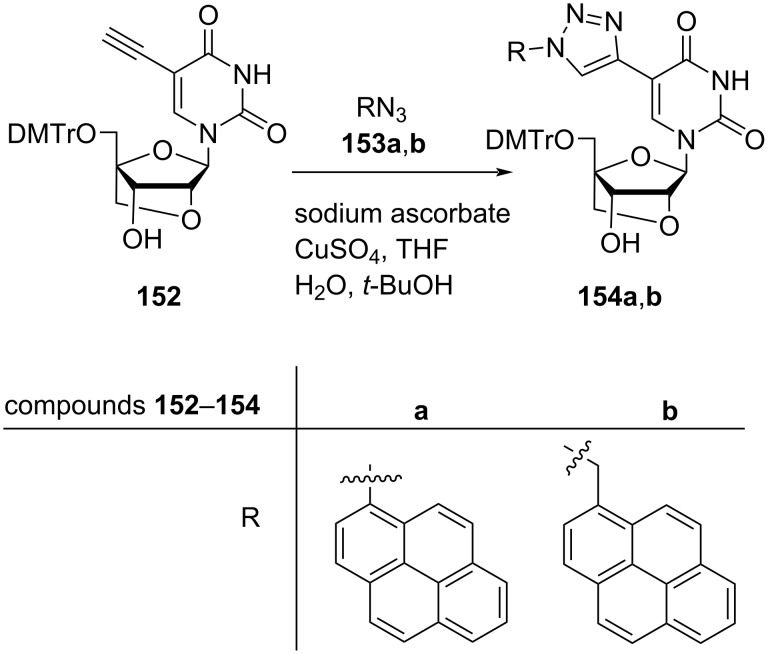
Synthesis of 5-*C*-triazolyl-functionalized double-headed nucleosides **154a**, **b**.

Hrdlicka and co-workers [24] also synthesized 5-*C*-triazolyl-functionalized double-headed nucleosides **154a**,**b** starting from 5-*C*-ethynyl-functionalized LNA uridine **152**. The LNA uridine **152** was reacted with 1-azidopyrene (**153a)** and 1-azidomethylpyrene (**153b)** separately under copper-catalyzed alkyne azide cycloaddition (CuAAC) reaction conditions to yield the double-headed nucleosides **154a** and **154b**, respectively (Scheme 39) [24].

The synthesized double-headed nucleosides **151a–d** and **154a**,**b** were phosphitylated, incorporated into oligonucleotides and characterized with respect to thermal denaturation, enzymatic stability, and fluorescence properties. The incorporation of the double-headed nucleosides **151a–d** and **154a**,**b** into oligonucleotides failed to form thermostable duplexes with complementary DNA and RNA strands but exhibited a potential resistance towards 3′-exonuclease. The synthesized double-headed nucleosides **151c**,**d** and **154a**,**b** when incorporated into oligonucleotides enabled fluorescent discrimination of targets with single nucleotide polymorphisms (SNPs) [24].

**Scheme 40:**
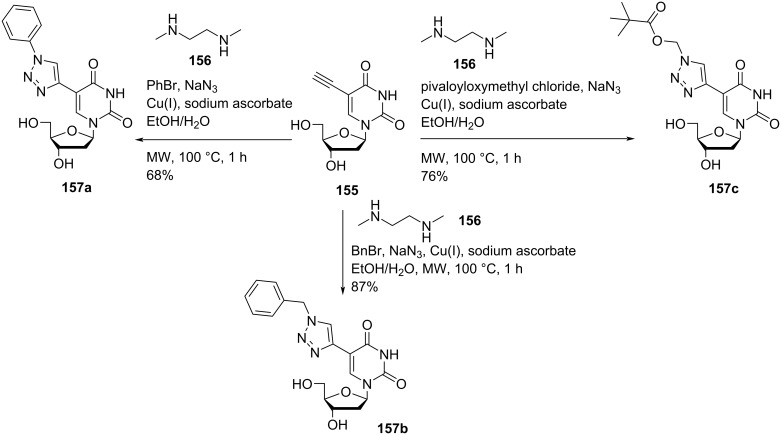
Synthesis of double-headed nucleosides **157a–c**.

Nielsen and co-workers [15] synthesized a series of double-headed nucleosides 5-(1-phenyl-1*H*-1,2,3-triazol-4-yl)-2′-deoxyuridine (**157a)**, 5-(1-benzyl-1*H*-1,2,3-triazol-4-yl)-2′-deoxyuridine (**157b**), and 5-(1-pivaloyloxymethyl-1*H*-1,2,3-triazol-4-yl)-2′-deoxyuridine (**157c**). The synthesis started from 5-ethynyl-2′-deoxyuridine (**155)** which in turn was synthesized from 5-iodo-2′-deoxyuridine following literature procedures [77–79]. The terminal alkyne **155** was reacted with bromobenzene and sodium azide under microwave heating in an EtOH/H_2_O mixture in the presence of copper iodide, sodium ascorbate, and *N*,*N*-dimethylethylenediamine (**156**) to afford the double-headed nucleoside **157a**. The reaction of the terminal alkyne **155** with benzyl bromide and pivaloyloxymethyl chloride under similar conditions afforded the double-headed nucleosides **157b** and **157c**, respectively (Scheme 40) [15].

The double-headed nucleosides **157a–c** were introduced into nonamer oligonucleotides by phosphoramidite chemistry [15]. a single incorporation of double-headed nucleosides **157a–c** into oligonucleotides resulted in the formation of unstable duplexes with complementary DNA and RNA strands whereas four consecutive incorporations led to increased duplex stability due to an efficient stacking of heteroaromatic triazoles as revealed by CD spectroscopy and molecular dynamics simulations [15,22]. The double-headed nucleoside **157a** was further used for the synthesis of 5-(phenyltriazol)-2′-deoxyuridine-modified 2′-*O*-methyl mixmer antisense oligonucleotides (AOs). The obtained AOs were investigated for their potential to induce exon skipping in DMD (Duchenne muscular dystrophy) transcript using *H2K mdx* mouse myotubes. It was found that exon-23 skipping potential of oligonucleotide containing 5-(phenyltriazole)-2′-deoxyuridine (**157a**) building blocks placed distantly was slightly better than oligonucleotides containing the 5-(phenyltriazole)-2′-deoxyuridine (**157a**) building blocks placed consecutively [80].

**Scheme 41:**
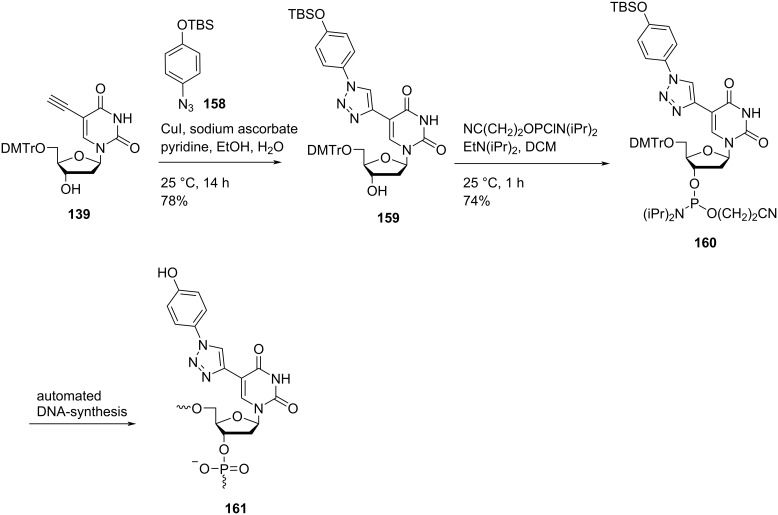
Synthesis of double-headed nucleoside **159**, phosphoramidite **160** and the corresponding nucleotide monomer **161**.

**Scheme 42:**
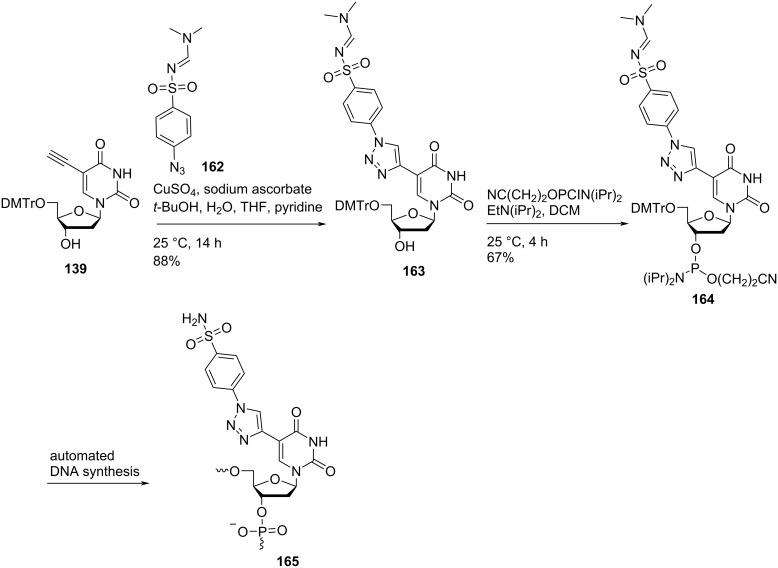
Synthesis of double-headed nucleoside **163**, phosphoramidite **164** and the corresponding nucleotide monomer **165**.

Nielsen and co-workers [26] synthesized triazole-containing double-headed nucleosides **159** and **163** by the reaction of 5′-*O*-dimethoxytritylated nucleoside **139** with *tert*-butyldimethylsilyl 4-azidophenylether (**158**) and *N*-(dimethylaminomethylidene)-4-azidobenzenesulfonamide (**162**), respectively under copper-catalyzed alkyne azide cycloaddition (CuAAC) reaction conditions (Scheme 41 and Scheme 42) [26].

The synthesized double-headed nucleosides **159** and **163** were reacted with 2-cyanoethyl-*N*,*N*-diisopropyl-phosphoramidochloridite in the presence of DIPEA (*N*,*N*-diisopropylethylamine) to afford phosphoramidites **160** and **164** which were then incorporated into oligodeoxynucleotides using automated solid phase synthesis. The synthesized oligonucleotides were removed from the solid support by treatment with concentrated aqueous ammonia which resulted in the formation of incorporated monomers **161** and **165** by simultaneous removal of *tert*-butyldimethylsilyl and amidine protecting groups, respectively (Scheme 41 and Scheme 42) [26].

The incorporation of the double-headed nucleosides **159** and **163** into oligonucleotides resulted in the formation of thermally stable DNA:RNA duplexes due to an efficient π–π stacking between two or more phenyltriazoles in the major groove. The more stable duplex was obtained when oligonucleotide containing monomer **165** was hybridized with the complementary RNA strand due to the best stacking shown by sulfonamide-substituted phenyltriazoles in the major groove [26–27]. Single incorporations of 5-*C*-triazolylbenzenesulfonamide-substituted monomer **165** at four positions within the gap region of RNase H gapmer antisense oligonucleotides (ASOs) reduced wild-type and mutant huntingtin mRNA in human patient fibroblasts. A structural model of the catalytic domain of human RNase H bound to ASO:RNA heteroduplexes was created which was utilized for explaining the activity and selectivity observations in cells and in the biochemical assays [81].

**Scheme 43:**
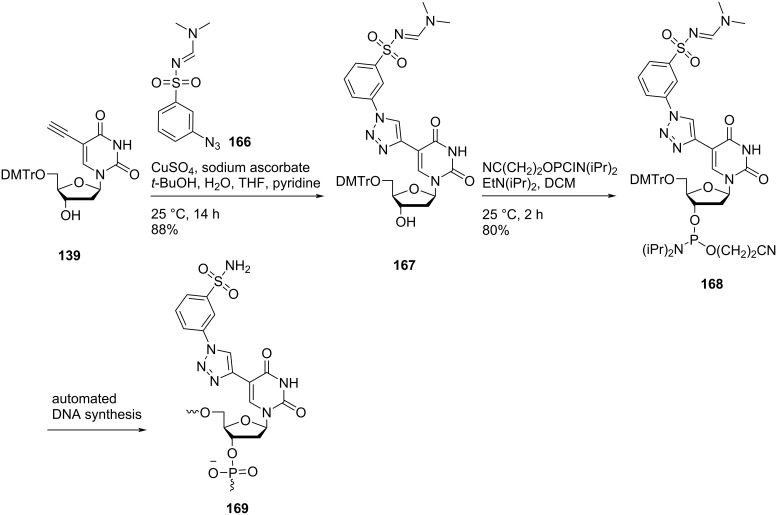
Synthesis of double-headed nucleoside **167**, phosphoramidite **168**, and the corresponding nucleotide monomer **169**.

Sharma and co-workers [27] synthesized the double-headed nucleoside **167** by reacting 5′-*O*-dimethoxytritylated nucleoside **139** with *N*-(dimethylaminomethylidene)-3-azidobenzenesulfonamide (**166)** under copper-catalyzed alkyne–azide cycloaddition (CuAAC) reaction conditions (Scheme 43). The synthesized double-headed nucleoside **167** was further reacted with 2-cyanoethyl-*N*,*N*-diisopropyl-phosphoramidochloridite in the presence of DIPEA to afford phosphoramidite **168** which was then incorporated into oligodeoxynucleotides using automated solid phase synthesis. The synthesized oligonucleotides were removed from the solid support by treatment with concentrated aqueous ammonia which resulted in the formation of incorporated monomer **169** by removal of the amidine protection (Scheme 43) [27].

**Scheme 44:**
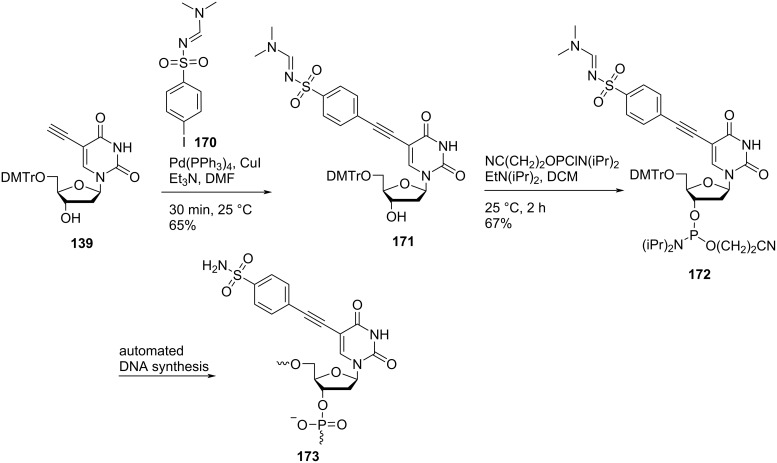
Synthesis of double-headed nucleoside **171**, phosphoramidite **172**, and the corresponding nucleotide monomer **173**.

**Scheme 45:**
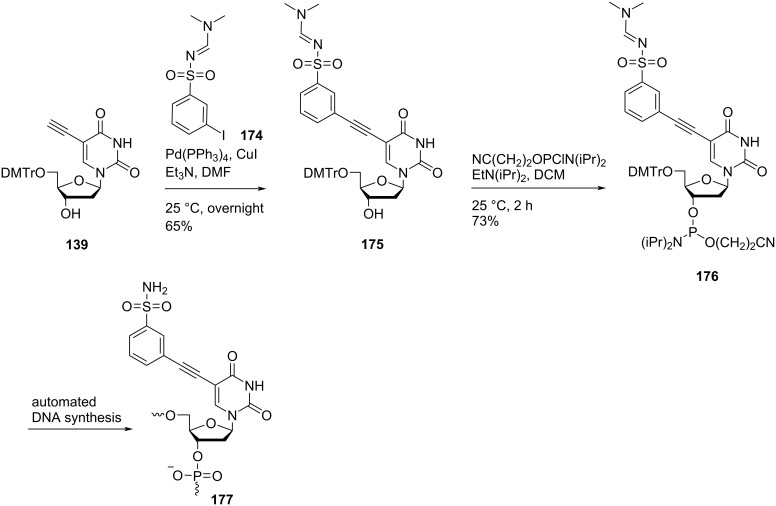
Synthesis of double-headed nucleoside **175**, phosphoramidite **176**, and the corresponding nucleotide monomer **177**.

Sharma and co-workers [27] also synthesized double-headed nucleosides **171** and **175** by the Sonogashira coupling of 5′-*O*-dimethoxytritylated alkyne **139** with *N*-(dimethylaminomethylidene)-4-iodobenzenesulfonamide (**170**) and *N*-(dimethylaminomethylidene)-3-iodobenzenesulfonamide (**174**), respectively (Scheme 44 and Scheme 45).

**Scheme 46:**
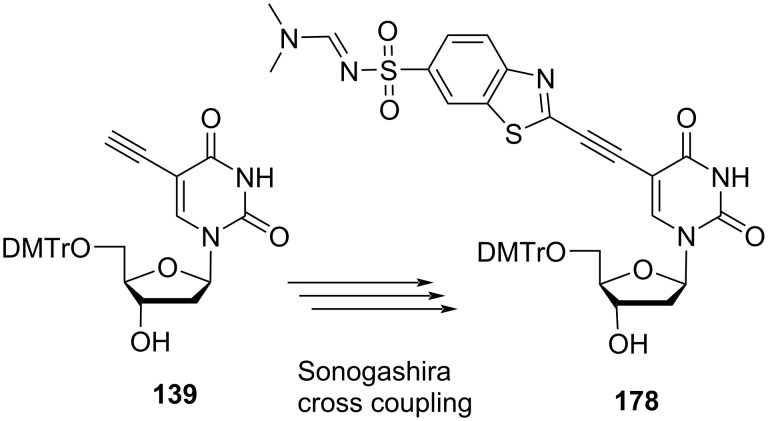
Synthesis of double-headed nucleoside **178**.

The synthesized double-headed nucleosides **171** and **175** were reacted with 2-cyanoethyl-*N*,*N*-diisopropyl-phosphoramidochloridite in the presence of DIPEA to afford phosphoramidites **172** and **176**, respectively. The phosphoramidites **172** and **176** were then incorporated into oligonucleotides using automated solid phase synthesis which after removal from the solid support by treatment with concentrated aqueous ammonia resulted in the formation of incorporated monomers **173** and **177**, respectively by removal of the amidine protection of sulfonamides (Scheme 44 and Scheme 45) [27]. The double-headed nucleoside **178** was also synthesized starting from 5′-*O*-dimethoxytritylated alkyne **139** under Sonogashira cross coupling reaction conditions (Scheme 46) [28].

The incorporation of the double-headed nucleoside **167** into oligonucleotides resulted in the formation of an equally stable DNA:RNA duplex as in the case of double-headed nucleoside **163** irrespective of the positional orientation of the sulfonamide group due to an efficient π–π stacking between two or more phenyltriazoles in the major groove [27]. On the other hand, the incorporation of the double-headed nucleosides **171** and **175** into oligonucleotides resulted in the formation of less stable DNA:RNA duplexes because of the poor stacking by the alkynyl group as compared to triazolyl groups in double-headed nucleosides **163** and **167** [27]. The double-headed nucleotide **173** was fully accepted by KOD (kodakaraensis), Phusion, and Klenow DNA polymerases as substrate which resulted in the formation of fully extended DNA. KOD DNA polymerase was found to be the best enzyme to produce DNA containing the double-headed nucleotide **173** in good yield and Phusion DNA polymerase amplified the template containing double-headed nucleotide **173** efficiently by PCR (polymerase chain reaction) [82].

**Scheme 47:**
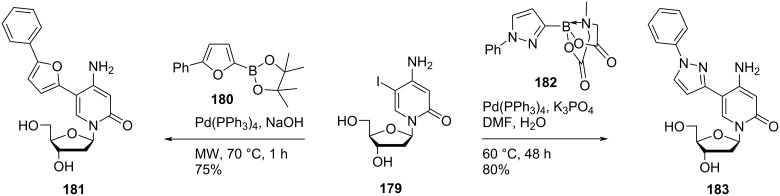
Synthesis of the double-headed nucleosides **181** and **183**.

Nielsen and co-workers [22] synthesized the double-headed nucleoside **181** by Suzuki–Miyaura cross coupling reaction of 5-iodo-2′-deoxycytidine (**179**) with 5-phenylfuran-2-boronic acid pinacol ester (**180**) in the presence of Pd(PPh_3_)_4_ and NaOH (Scheme 47).

They also synthesized the double-headed nucleoside **183** by K_3_PO_4_-mediated [83] Suzuki–Miyaura cross coupling reaction of 5-iodo-2′-deoxycytidine (**179**) with pyrazole MIDA (*N*-methyliminodiacetic acid) boronate **182** which in turn was synthesized by sydnone–alkyne cycloaddition reaction between ethynylboronic acid MIDA ester and *N*-phenylsydnone (Scheme 47) [22].

**Scheme 48:**
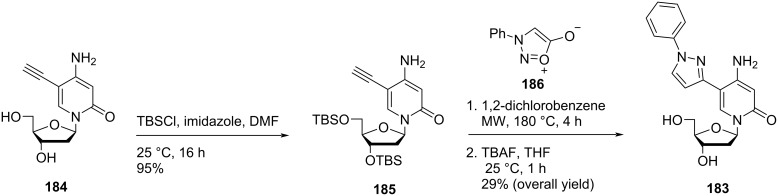
Alternative synthesis of the double-headed nucleoside **183**.

The double-headed nucleoside **183** was also synthesized from 5-ethynyl-2′-deoxycytidine (**184**) which was first converted into 3′,5′-bis-*O*-(*tert*-butyldimethylsilyl)-5-ethynyl-2′-deoxycytidine (**185**). The protected nucleoside **185** was reacted with *N*-phenylsydnone (**186**) via thermal [2 + 3] sydnone–alkyne cycloaddition [84] to afford the double-headed nucleoside **183** (Scheme 48) [22].

**Scheme 49:**
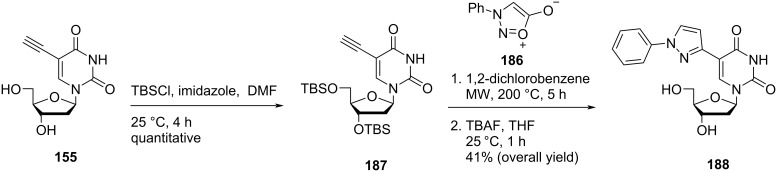
Synthesis of double-headed nucleoside **188** through thermal [2 + 3] sydnone–alkyne cycloaddition reaction.

Nielsen and co-workers [22] also synthesized the double-headed nucleoside **188** via thermal [2 + 3] sydnone–alkyne cycloaddition reaction between 3′,5′-bis-*O*-(*tert*-butyldimethylsilyl)-5-ethynyl-2′-deoxyuridine (**187**) and *N*-phenylsydnone (**186**) (Scheme 49).

The incorporation of the double-headed nucleosides **181** and **183** multiple times into oligonucleotides resulted in the formation of stable DNA:RNA duplexes due to the perfect stacking of the aromatic moieties in the major groove of the duplex [22]. The double-headed nucleoside **183** containing a phenylpyrazole moiety exhibited better π–π stacking interactions in the major groove with itself and with an adjacent double-headed nucleoside (**157a**) incorporated as compared to the double-headed nucleoside **181** containing a flexible phenylfuran moiety. There was not any change in the geometry of the duplexes observed upon introduction of double-headed nucleosides **181** and **183** as revealed by CD spectroscopy and molecular modeling. The synthesized oligonucleotides containing consecutive triazole-functionalized double-headed nucleosides **183** and **157a** were found to form highly stable duplexes due to a large aromatic overlap of their substituents at the 5-position due to which they can be utilized as a simple tool in high affinity RNA targeting oligonucleotides [22].

**Scheme 50:**
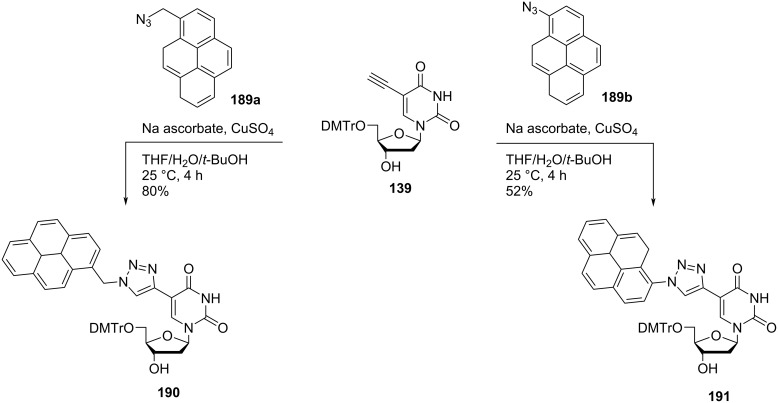
Synthesis of the double-headed nucleosides **190** and **191**.

Hrdlicka and co-workers [25] synthesized the double-headed nucleosides **190** and **191** starting from *C*5-ethynyl-5′-*O*-(4,4′-dimethoxytrityl)-2′-deoxyuridine (**139**) which in turn was synthesized from 5-iodo-2′-deoxyuridine following a procedure reported in the literature [77]. The nucleoside **139** was reacted with 1-azidomethylpyrene (**189a**) and 1-azidopyrene (**189b**) under copper-catalyzed alkyne–azide cycloaddition (CuAAC) reaction conditions to afford the double-headed nucleosides **190** and **191**, respectively (Scheme 50) [25].

The incorporation of the double-headed nucleoside **190** into oligonucleotides displayed significant hybridization-induced increase in fluorescence emission whereas the double-headed nucleoside **191** allowed for efficient fluorescent discrimination of SNPs (single nucleotide polymorphisms) via a G-specific quenching mechanism when incorporated into oligonucleotides [25].

### Pyranosyl double-headed nucleosides

Synthetic methodologies have been developed for placing the additional nucleobase at various positions in the pyranonucleosides. Here, we have categorized the double-headed pyranosyl nucleoside monomers depending on the point of attachment at the pyranose sugar. The double-headed pyranosyl nucleosides have the nucleobase attached at the C-6′ position of the pyranose moiety, either directly or by a methylene linker (Figure 1). Pyranosyl nucleosides where the additional nucleobase is attached at the C-3′ position of the pyranosyl moiety through a triazolo-linker have also been synthesized.

**Scheme 51:**
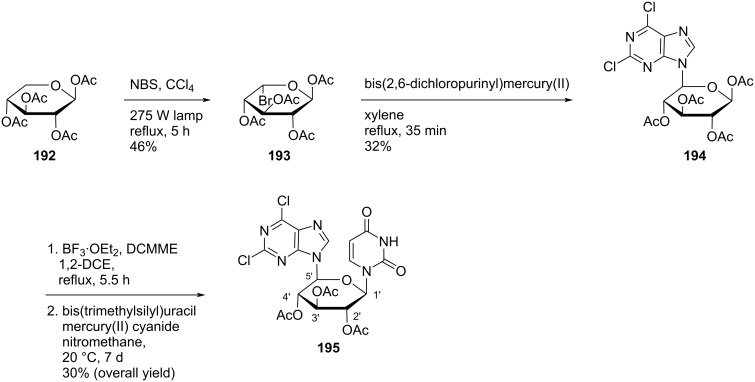
Synthesis of 1-((5*S*)-2,3,4-tri-*O*-acetyl-5-(2,6-dichloropurin-9-yl)-β-ᴅ-xylopyranosyl)uracil (**195**).

Ferrier and Tyler [85] synthesized the double-headed nucleoside 1-[(5*S*)-2,3,4-tri-*O*-acetyl-5-(2,6-dichloropurin-9-yl)-β-ᴅ-xylopyranosyl]uracil (**195**) by photobromination of tetra-*O*-acetyl-β-ᴅ-xylopyranose (**192**) to afford the crystalline product **193** which upon reaction with bis(2,6-dichloropurinyl)mercury in xylene afforded crystalline compound **194**. Subsequently, the nucleoside analogue **194** was reacted with BF_3_·OEt_2_ followed by reaction with silylated uracil to get the double-headed nucleoside **195** (Scheme 51) [85].

**Scheme 52:**
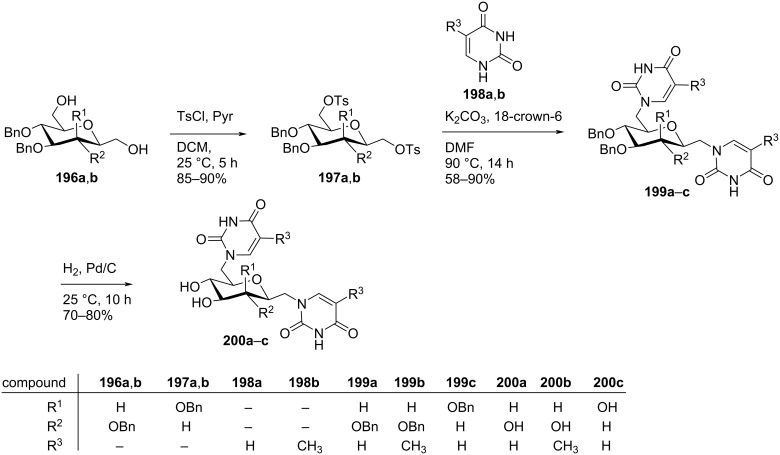
Synthesis of hexopyranosyl double-headed pyrimidine homonucleosides **200a–c**.

Prasad and co-workers [86] synthesized hexopyranosyl double-headed pyrimidine homonucleosides 1-[(6-deoxy-6-(uracil-1-yl)-β-ᴅ-glucopyranosyl)methyl]uracil (**200a**), 1-[(6-deoxy-6-(thymin-1-yl)-β-ᴅ-2,3,4-tri-*O*-benzylglucopyranosyl)methyl]thymine (**200b**) and 1-[(6-deoxy-6-(uracil-1-yl)-β-ᴅ-mannopyranosyl)methyl]uracil (**200c**) from dihydroxy 2,6-anhydro-3,4,5-tri-*O*-benzylheptitols (**196a**,**b**) which in turn were synthesized from ᴅ-glucose and ᴅ-mannose [87]. The benzylated 2,6-anhydroheptitols **196a**,**b** were reacted with tosyl chloride to form the ditosylated compounds **197a**,**b** which upon reaction with substituted thymine and uracil **198a**,**b** afforded the benzylated double-headed nucleosides **199a**–**c**. Next, debenzylation of the nucleoside monomers **199a–c** afforded the final double-headed nucleoside monomers **200a–c**. (Scheme 52) [86].

**Figure 2:**
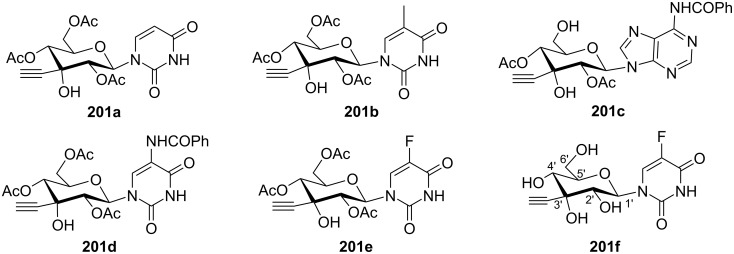
3′-*C*-Ethynyl-β-ᴅ-allopyranonucleoside derivatives **201a–f**.

**Scheme 53:**
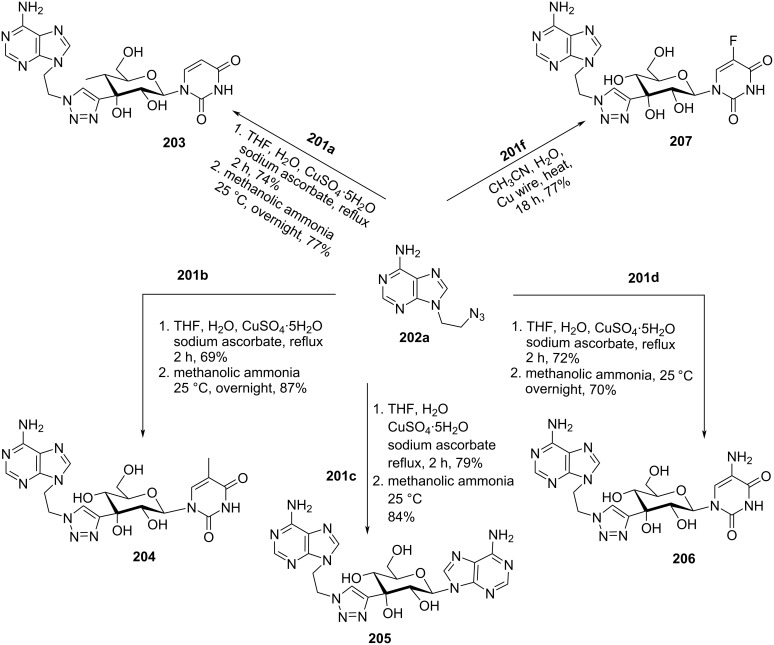
Synthesis of 3′-*C*-(1,4-disubstituted-1,2,3-triazolyl)-double-headed pyranonucleosides **203**–**207**.

**Scheme 54:**
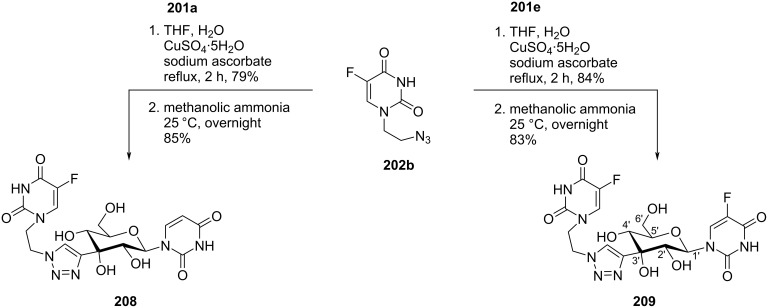
Synthesis of 3′-*C*-(1,4-disubstituted-1,2,3-triazolyl)-double-headed pyranonucleosides **208** and **209**.

**Scheme 55:**
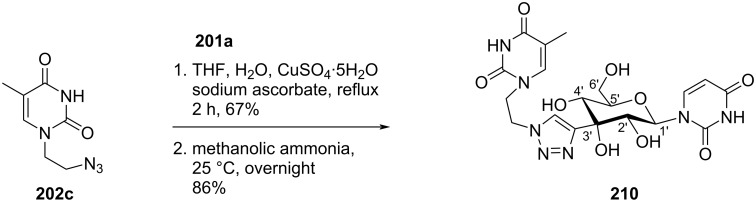
Synthesis of 3′-*C*-(1,4-disubstituted-1,2,3-triazolyl)-double-headed pyranonucleoside **210**.

Komiotis and co-workers [37] synthesized 3′-*C*-(1,4-disubstituted-1,2,3-triazolyl)-substituted double-headed pyranonucleosides **203–210** from 3′-*C*-ethynyl-β-ᴅ-allopyranonucleoside derivatives **201a–f** (Figure 2) which in turn were synthesized from 1,2:5,6-di-*O*-isopropylidene-α-ᴅ-ribohexofuranos-3-ulose [88]. The 3′-*C*-ethynyl-substituted pyranonucleoside derivatives **201a–f** were reacted with azidoethyladenine, 5-fluorouracil and thymine **202a–c** via copper-catalyzed azide–alkyne cycloaddition (CuAAC) reaction followed by treatment with methanolic ammonia to afford the double-headed nucleosides **203–210** (Scheme 53, Scheme 54, and Scheme 55) [37].

The double-headed nucleosides **203–210** were evaluated for their antiviral and cytostatic activities, and the nucleosides **204**, **206**, and **207** showed moderate cytostatic activity against human cervix carcinoma HeLa cells [37].

### Acyclic double-headed nucleosides

In this section, double-headed nucleosides are included that have an acyclic carbohydrate moiety and the heterocyclic moieties/nucleobases are terminally attached at the sugar moiety (Figure 1).

**Scheme 56:**
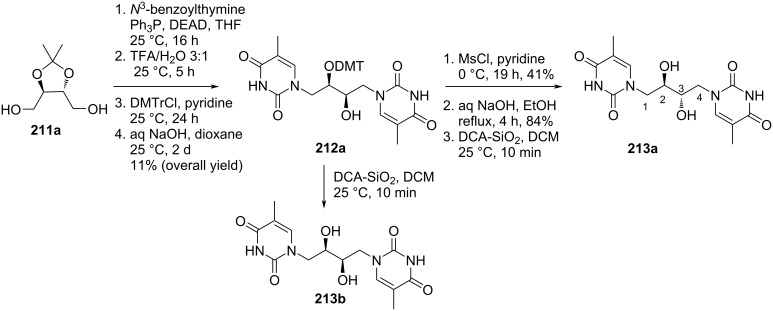
Synthesis of double-headed acyclic nucleosides (2*S*,3*R*)-1,4-bis(thymine-1-yl)butane-2,3-diol (**213a**) and (2*R*,3*R*)-1,4-bis(thymine-1-yl)butane-2,3-diol (**213b**).

**Scheme 57:**
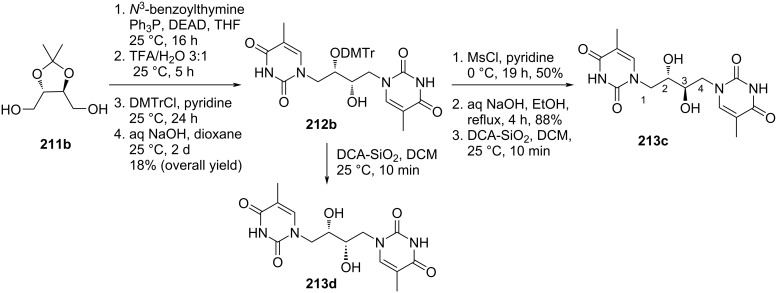
Synthesis of double-headed acyclic nucleosides (2*R*,3*S*)-1,4-bis(thymine-1-yl)butane-2,3-diol (**213c**) and (2*S*,3*S*)-1,4-bis(thymine-1-yl)butane-2,3-diol (**213d**).

Nielsen and co-workers [89] synthesized four stereoisomers of double-headed acyclic nucleosides 1,4-bis(thymine-1-yl)butane-2,3-diols **213a–d** starting from either ᴅ- or ʟ-2,3-*O*-isopropylidenethreitol **211a**,**b**. The dihydroxy compounds **211a**,**b** were reacted with *N*^3^-benzoylthymine under Mitsunobu reaction conditions followed by DMTr protection to give two enantiopure compounds (2*R*,3*R*)-1,4-bis(thymin-1-yl)-3-*O*-DMTr-butan-2-ol (**212a)** and (2*S*,3*S*)-1,4-bis(thymin-1-yl)-3-*O*-DMTr-butan-2-ol (**212b**). The two compounds upon removal of the DMTr group gave the acyclic double-headed nucleosides (2*R*,3*R*)-1,4-bis(thymine-1-yl)butane-2,3-diol (**213b**) and (2*S*,3*S*)-1,4-bis(thymine-1-yl)butane-2,3-diol (**213d**). Further, the reaction of the 3-*O*-DMTr-protected nucleosides **212a**,**b** with mesyl chloride followed by treatment with aq. NaOH in ethanol afforded the nucleosides (2*S*,3*R*)-1,4-bis(thymine-1-yl)butane-2,3-diol (**213a**) and (2*R*,3*S*)-1,4-bis(thymine-1-yl)butane-2,3-diol (**213c) (**Scheme 56 and Scheme 57) [89].

These double-headed nucleosides when incorporated into oligonucleotides destabilized both DNA and RNA duplexes. However, nucleosides with 2′(*S*)-configuration were found to destabilize duplexes and bulged motifs to a lesser extent than the other stereoisomers [89].

**Scheme 58:**
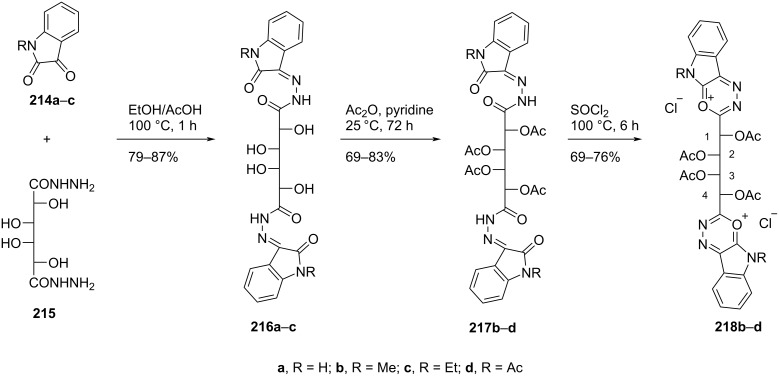
Synthesis of double-headed acetylated 1,3,4-oxadiazino[6,5-*b*]indolium-substituted *C*-nucleosides **218b**–**d**.

Nasr [16] synthesized 1,4-bis(9-methyl-1,3,4-oxadiazino[6,5-*b*]indol-2-yl-1-ium) dichloride, 1,4-bis(9-ethyl-1,3,4-oxadiazino[6,5-*b*]indol-2-yl-1-ium) dichloride, and 1,4-bis(9-acetyl-1,3,4-oxadiazino[6,5-*b*]indol-2-yl-1-ium) dichloride-substituted double-headed nucleosides of 1,2,3,4-tetra-*O*-acetylgalactotetritol **218b–d** starting from 1,3-dihydro-2,3-dioxo-2*H*-indoles **214a–c**. The indoles were condensed with galactaric acid bishydrazide to give compounds **216a–c** which upon acetylation followed by heterocyclization in the presence of thionyl chloride afforded nucleosides **218b–d** (Scheme 58) [16].

The synthesized double-headed nucleosides **218b–d** may exhibit potential biological activities due to the resistance of the C-glycosidic moiety towards hydrolytic or enzymatic cleavage [90] and the enhanced hydrophilicity which results in an increased transportation to biological systems [16].

**Scheme 59:**
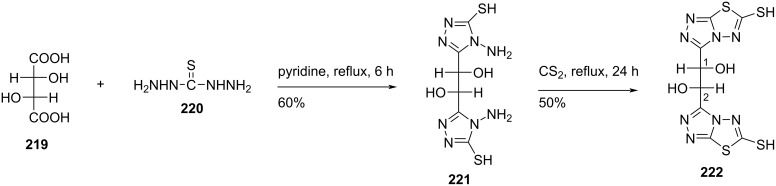
Synthesis of double-headed acyclic nucleoside **222**.

**Scheme 60:**
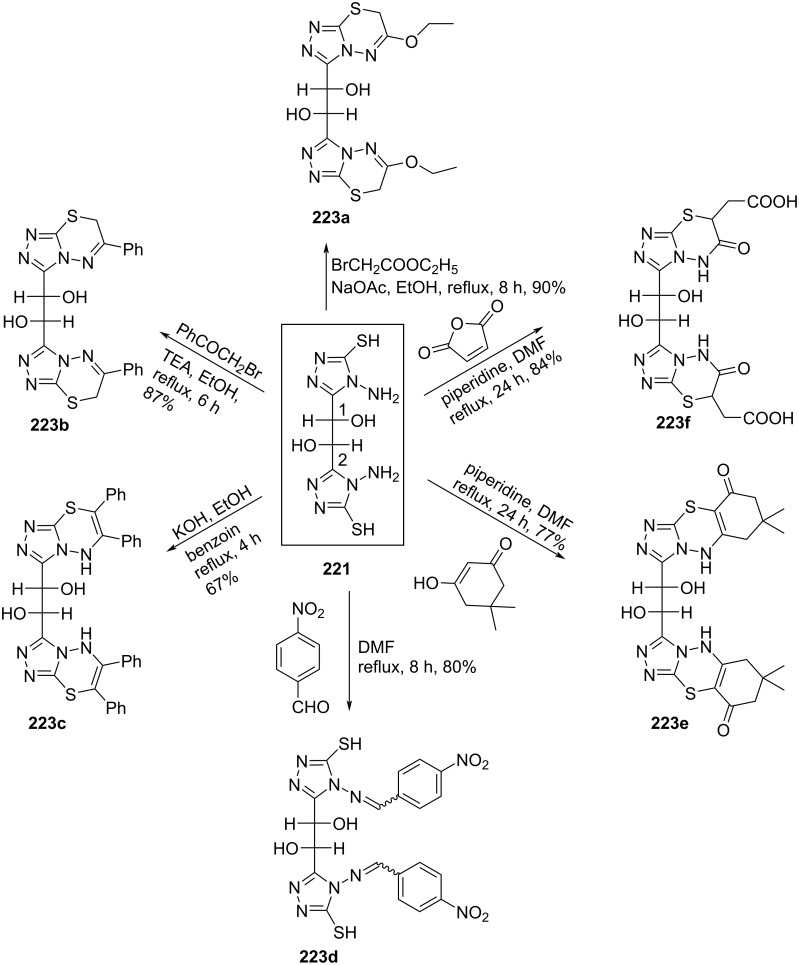
Synthesis of functionalized 1,2-bis(1,2,4-triazol-3-yl)ethane-1,2-diols **223a–f**.

El Ashry and co-workers [17] synthesized functionalized 1,2-bis(1,2,4-triazol-3-yl)ethane-1,2-diols **222** and **223a–f** starting from (1*R*,2*S*)-1,2-bis(4-amino-5-mercapto-1,2,4-triazol-3-yl)-ethane-1,2-diol (**221**) which in turn was synthesized by reacting ʟ-tartaric acid (**219**) with thiocarbohydrazide. The reaction of 4-amino-5-mercapto-3-substituted-1,2,4-triazole **221** with carbon disulfide, ethyl bromoacetate, phenacyl bromide, benzoin, *p*-nitrobenzaldehyde, dimedone, and maleic anhydride afforded the double-headed nucleosides **222** and **223a–f** (Scheme 59 and Scheme 60) [17].

The double-headed nucleosides **222** and **223a–f** were synthesized with the aim to evaluate their biological activities due to the potent inhibitory effect of the precursor 4-amino-5-mercapto-1,2,4-triazole against glycosidase enzymes [17,91–93].

**Scheme 61:**
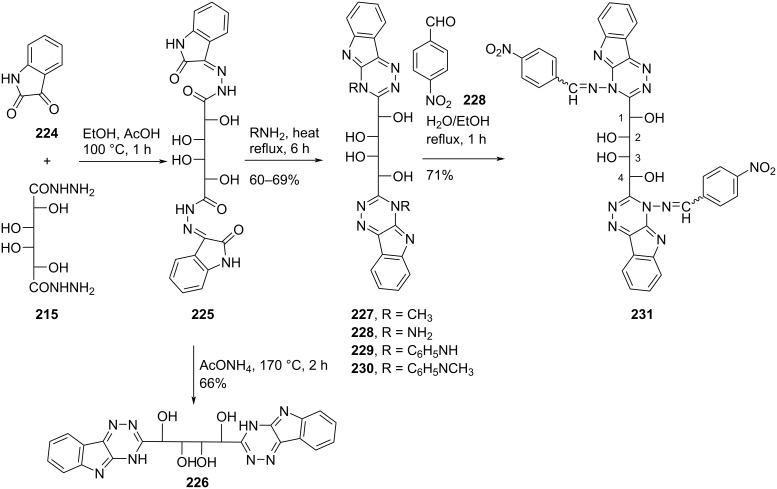
Synthesis of acyclic double-headed 1,2,4-triazino[5,6-*b*]indole *C*-nucleosides **226**–**231**.

Nasr [18] synthesized the double-headed acyclic 1,2,4-triazino[5,6-*b*]indole *C*-nucleosides **226–231** through the heterocyclization of bis(2-oxoindolin-3-ylidene)galactaric acid hydrazide (**225**) with various one-nitrogen cyclizing agents (Scheme 61).

The synthesized double-headed nucleosides **226–231** were expected to possess potent biological activities due to the known antimicrobial [94–98], antiviral [99], antihypertensive [99–100], analgesic [101], and antitumor activities [102] exhibited by various derivatives of 1,2,4-triazino[5,6-*b*]indole [18].

**Scheme 62:**
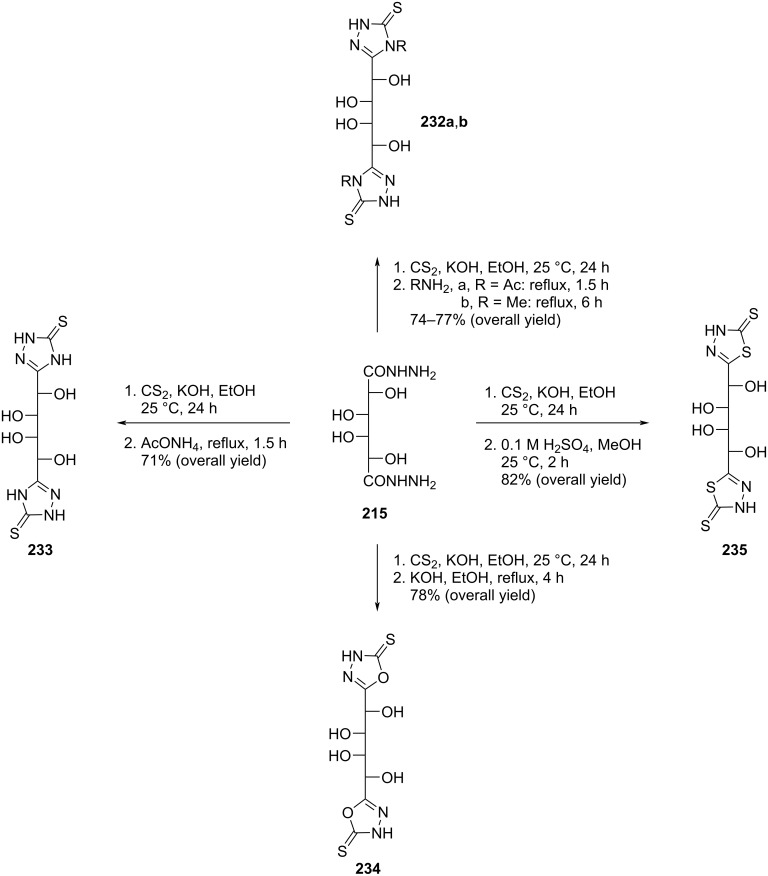
Synthesis of double-headed 1,3,4-thiadiazoline, 1,3,4-oxadiazoline, and 1,2,4-triazoline acyclo *C*-nucleosides **232a**,**b** and **233**–**235**.

Nasr and co-workers [19] also synthesized double-headed 1,2,4-triazoline (**232a,b**, **233**), 1,3,4-oxadiazoline (**234**), 1,3,4-thiadiazoline (**235**) acyclo *C*-nucleosides starting from galactaric acid hydrazide (**215**). The syntheses started with the condensation of compound **215** with carbon disulfide in the presence of ethanolic potassium hydroxide to give the dipotassium salt of galactaric acid bis(hydrazidocarbodithioic acid) which was then heterocyclized under different reaction conditions to give three types of double-headed nucleosides (Scheme 62) [19].

The acyclic double-headed nucleosides **232a** and **233–235** were screened for their in vitro antibacterial activity against the Gram-negative bacterium *Escherichia coli* and the Gram-positive bacterium *Staphylococcus aureus* and for their antifungal activity against *Candida albicans* using the agar diffusion method [103]. Among the tested compound, derivative **235** showed fair activity against *E. coli* and *C. albicans* but was inactive against *S. aureus* whereas compound **234** showed activity only against *S. aureus* [19].

**Scheme 63:**
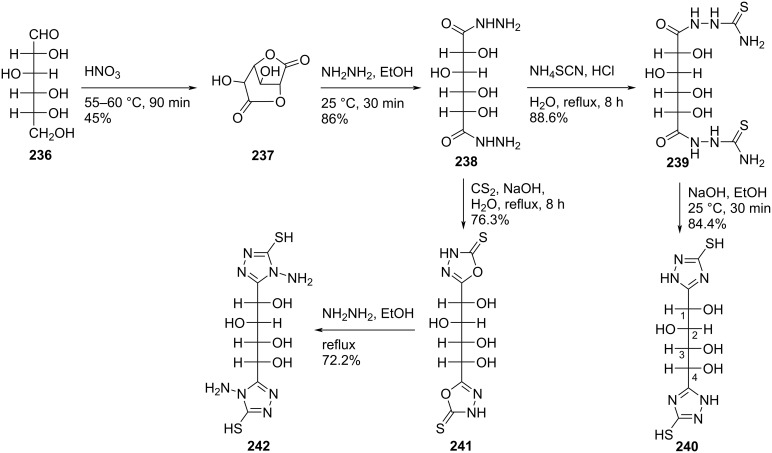
Synthesis of double-headed acyclo *C*-nucleosides **240**–**242**.

Amara and Othman [20] synthesized the double-headed acyclo-*C*-nucleosides 1,4-bis(3-mercapto-1*H*-1,2,4-triazol-5-yl)butane-1,2,3,4-tetrol **240**, 5,5′-(1,2,3,4-tetrahydroxybutane-1,4-diyl)-bis(1,3,4-oxadiazole-2(3*H*)-thione) **241**, and 1,4-bis(4-amino-5-mercapto-4*H*-1,2,4-triazol-3-yl)butane-1,2,3,4-tetrol **242** starting from ᴅ-glucose (**236**). The inexpensive sugar **236** was converted into 2,3,4,5-tetrahydroxyhexanedihydrazide **238** in two steps which was further reacted with either ammonium thiocyanate or carbon disulfide to give the bishydrazinocarbothioamide **239** and the acyclic double-headed nucleoside **241**, respectively. The double-headed nucleosides **240** and **242** were obtained by treatment of compound **239** with NaOH and of compound **241** with hydrazine hydrate, respectively (Scheme 63) [20].

The double-headed *C*-nucleosides **240**–**242** were tested in vitro against Gram-positive bacteria *Staphylococcus aureus*, *Listeria inovanii* and Gram-negative bacteria *Klebsiella pneumoniae*, *Salmonella* sp., and *Escherichia coli*. All the double-headed nucleosides except derivative **242** showed moderate antibacterial activity in comparison with the known antibiotic combination amoxicillin/clavulanic acid (AMC) [20].

The structural and electronic properties of the double-headed nucleosides were explored theoretically by performing semi-empirical molecular orbital, ab initio Hartree–Fock (HF), and density functional theory (DFT) calculations and their geometries were optimized at the level of Austin Model 1 (AM1) [104].

**Scheme 64:**
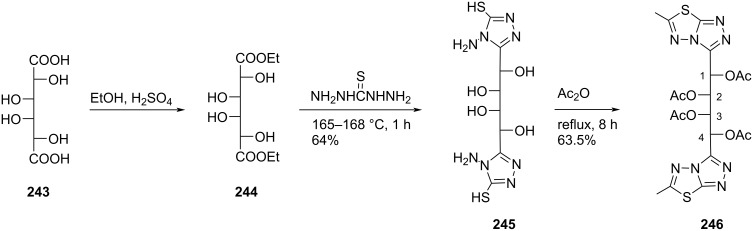
Synthesis of double-headed acyclo *C*-nucleoside **246**.

Galactaric acid (**243**) was diesterified with ethanol in the presence of conc. sulfuric acid. The corresponding diethyl ester **244** was treated with thiocarbohydrazide in a fusion reaction to produce compound **245**, which upon treatment with acetic anhydride under heating conditions, afforded the acyclic double-headed acyclic *C*-nucleoside **246** (Scheme 64) [21].

Some 3,6-disubstituted 1,2,4-triazolo[3,4-*b*]1,3,4-thiadiazole derivatives possess anti-HIV-I [105], anti-inflammatory [106–107], anticancer [108–109], and antibacterial properties [110]. The double-headed *C*-nucleoside **246** comprises an alditolyl moiety attached at position 3 of the 1,2,4-triazolo[3,4-*b*]1,3,4-thiadiazole core which can enhance the biological activity due to the hydrophilic nature of the alditolyl residue which may further increase the transportation into biological systems [21].

**Scheme 65:**
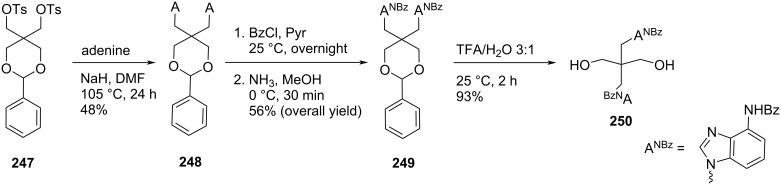
Synthesis of acyclo double-headed nucleoside **250**.

Compound **247** was treated with adenine in the presence of sodium hydride in DMF at 105 °C to incorporate two adenine moieties affording compound **248**. The benzoylation of compound **248**, followed by treatment with methanolic ammonia at low temperature produced the corresponding *N*-benzoylated adenine derivative **249** [111]. The cleavage of the diacetal in compound **249** was achieved with 75% TFA/water resulting in compound **250**, which was considered as the acyclic double-headed nucleoside without any protection of the primary hydroxy groups (Scheme 65) [111].

**Scheme 66:**
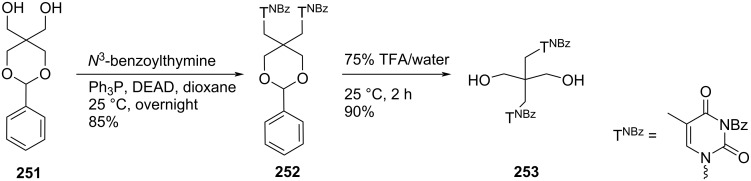
Synthesis of acyclo double-headed nucleoside **253**.

In a similar reaction sequence, compound **251** was treated with *N*^3^-benzoylthymine to afford compound **252**, which was treated with 75% TFA–water for deprotection of the hydroxy groups to afford the final monomer **253** (Scheme 66) [111].

The synthesized acyclo nucleosides **250** and **253** were phosphitylated and incorporated into oligonucleotides to evaluate the effects on duplex stability. It was observed that the hybridization properties of the oligonucleotides with one acyclic achiral nucleoside, i.e., **250** or **253** when incorporated in the middle of a 12-mer or 13-mer decreased with complementary DNA or RNA [111].

**Scheme 67:**
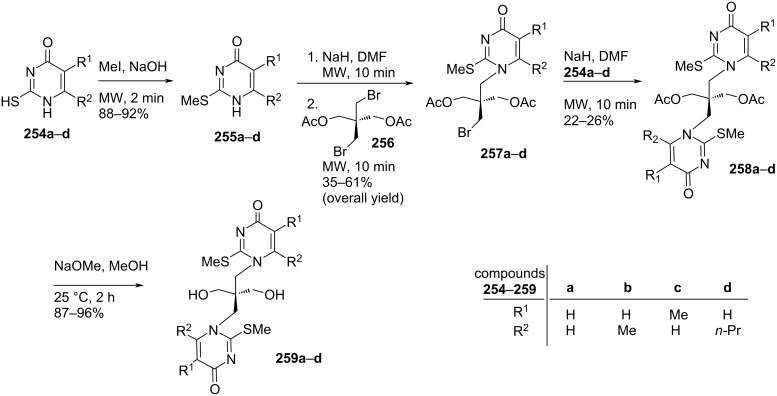
Synthesis of acyclo double-headed nucleosides **259a–d**.

Four pyrimidine nucleobases **254a–d** were treated with methyl iodide in the presence of sodium hydroxide to get methylthio derivatives **255a–d**, which were treated with 2,2-bis(bromomethyl)-1,3-diacetoxypropane (**256**) in the presence of NaH in DMF to afford the mono-headed acyclic nucleosides **257a–d** [112]. The second nucleobase was introduced in compounds **257a**–**d** by repeating the reaction with the desired nucleobase under otherwise identical conditions (NaH/DMF) giving the acyclic double-headed pyrimidine nucleosides **258a–d**. Finally, the treatment of compounds **258a**–**d** with NaOMe in methanol produced the unprotected nucleosides **259a–d** (Scheme 67) [112].

**Scheme 68:**
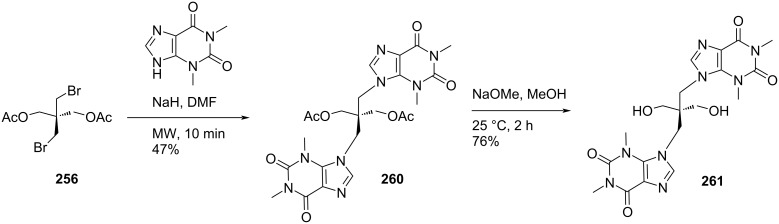
Synthesis of acyclo double-headed nucleoside **261**.

The double-headed nucleoside **261** was obtained by a two-step reaction sequence starting from compound **256**, which was first reacted with theophylline in DMF, to give the acyclic double-headed purine nucleoside **260** followed by treatment with NaOMe in methanol to get the unprotected product **261**. In the sequence, all reactions were carried out under microwave irradiation conditions (Scheme 68) [112].

The branched chain tetraseco-nucleosides **259a–d** and **261** were synthesized because acyclic nucleosides of tetraseco-type were found to possess interesting antiviral activities [21,113–115].

## Conclusion

Among the variety of modified nucleosides, double-headed nucleoside monomers are an important class of compounds, which have shown their importance in nucleoside chemistry. Here, we have focused on the available methodologies for the synthesis of several double-headed nucleosides. For a systematic discussion, we have classified them into three different categories, i.e., double-headed nucleosides with additional head/nucleobase on the sugar moieties, nucleobase moieties and on acyclic carbohydrate moieties. We have subdivided the category of monocyclic furanosyl double-headed nucleosides into 1,2-furanosyl-, 1,3-furanosyl-, 1,4-furanosyl-, and 1,5-furanosyl double-headed nucleosides depending on the position of the aglycon moiety in the furanosyl ring and systematically described their synthetic methodologies. Next, we elaborated the procedures for the synthesis of bicyclic furanosyl double-headed nucleosides, followed by procedures for the development of base to base double-headed nucleosides. The chemical strategies for the synthesis of pyranosyl double-headed nucleosides and acyclic double-headed nucleosides were also described. Along with the methodologies for the development of double-headed nucleoside monomers, the synthetic approach for their incorporation into the oligonucleotides was also elaborated in this review. Biological applications of the synthesized nucleosides were also described.

## Future Direction

Double-headed nucleosides are important structural scaffolds that modulate nucleic acid structures. Rationally designed nucleosides can tune interstrand and intrastrand interactions that are exhibited in nucleic acids. As a consequence, these synthetic scaffolds can be exploited rationally in biomolecular designs and medicinal chemistry. These modified double-headed nucleosides could be incorporated into oligonucleotides to explore their potential as antisense nucleosides. Similarly, as some of these nucleosides have shown their potential as antimicrobial agents, they could be explored extensively for their biological activity. This review will help researchers to get an insight into the available procedures for the synthesis of double-headed nucleosides and briefly explores their role in modulating nucleic acid structures and in medicinal chemistry. The researchers working in the field of modified nucleosides will be encouraged further to take up challenges for the synthesis of currently unexplored double-headed nucleosides with extensive configurations, connectivity through different linkers, and exploration of different purine and pyrimidine moieties as nucleobases.
